# Systems approaches reveal that ABCB and PIN proteins mediate co-dependent auxin efflux

**DOI:** 10.1093/plcell/koac086

**Published:** 2022-03-18

**Authors:** Nathan L Mellor, Ute Voß, Alexander Ware, George Janes, Duncan Barrack, Anthony Bishopp, Malcolm J Bennett, Markus Geisler, Darren M Wells, Leah R Band

**Affiliations:** Division of Plant and Crop Sciences, School of Biosciences, University of Nottingham, Sutton Bonington Campus, Loughborough, LE12 5RD, UK; Division of Plant and Crop Sciences, School of Biosciences, University of Nottingham, Sutton Bonington Campus, Loughborough, LE12 5RD, UK; Division of Plant and Crop Sciences, School of Biosciences, University of Nottingham, Sutton Bonington Campus, Loughborough, LE12 5RD, UK; Division of Plant and Crop Sciences, School of Biosciences, University of Nottingham, Sutton Bonington Campus, Loughborough, LE12 5RD, UK; Division of Plant and Crop Sciences, School of Biosciences, University of Nottingham, Sutton Bonington Campus, Loughborough, LE12 5RD, UK; Division of Plant and Crop Sciences, School of Biosciences, University of Nottingham, Sutton Bonington Campus, Loughborough, LE12 5RD, UK; Division of Plant and Crop Sciences, School of Biosciences, University of Nottingham, Sutton Bonington Campus, Loughborough, LE12 5RD, UK; Department of Biology, University of Fribourg, Fribourg CH-1700, Switzerland; Division of Plant and Crop Sciences, School of Biosciences, University of Nottingham, Sutton Bonington Campus, Loughborough, LE12 5RD, UK; Division of Plant and Crop Sciences, School of Biosciences, University of Nottingham, Sutton Bonington Campus, Loughborough, LE12 5RD, UK; Centre for Mathematical Medicine and Biology, School of Mathematical Sciences, University of Nottingham, Nottingham NG7 2RD, UK

## Abstract

Members of the B family of membrane-bound ATP-binding cassette (ABC) transporters represent key components of the auxin efflux machinery in plants. Over the last two decades, experimental studies have shown that modifying ATP-binding cassette sub-family B (*ABCB*) expression affects auxin distribution and plant phenotypes. However, precisely how ABCB proteins transport auxin in conjunction with the more widely studied family of PIN-formed (PIN) auxin efflux transporters is unclear, and studies using heterologous systems have produced conflicting results. Here, we integrate ABCB localization data into a multicellular model of auxin transport in the *Arabidopsis thaliana* root tip to predict how ABCB-mediated auxin transport impacts organ-scale auxin distribution. We use our model to test five potential ABCB–PIN regulatory interactions, simulating the auxin dynamics for each interaction and quantitatively comparing the predictions with experimental images of the DII-VENUS auxin reporter in wild-type and *abcb* single and double loss-of-function mutants. Only specific ABCB–PIN regulatory interactions result in predictions that recreate the experimentally observed DII-VENUS distributions and long-distance auxin transport. Our results suggest that ABCBs enable auxin efflux independently of PINs; however, PIN-mediated auxin efflux is predominantly through a co-dependent efflux where co-localized with ABCBs.

## Teaser

Predicting the experimentally observed root-tip auxin distribution requires ABCBs to efflux auxin independently, whereas PINs predominantly mediate auxin efflux where co-localized with ABCBs.

## Introduction

The plant hormone auxin plays an integral role in plant growth and regulates many aspects of plant development including organ initiation and tropic responses (Benjamins and Scheres, 2008). These processes have been studied in detail in the model species *Arabidopsis thaliana* where it has been shown that they depend on the spatial auxin distribution, which is controlled at the subcellular scale by the presence and localization of efflux and influx transporters on the cell membranes (Reinhardt et al., 2003). Determining how the transporters control the organ-scale auxin dynamics is essential to understanding auxin-related phenotypes, and mathematical and computational models have proven to be vital in interpreting experimental results (Krupinski and Jönsson, 2010).

**Figure 1:**
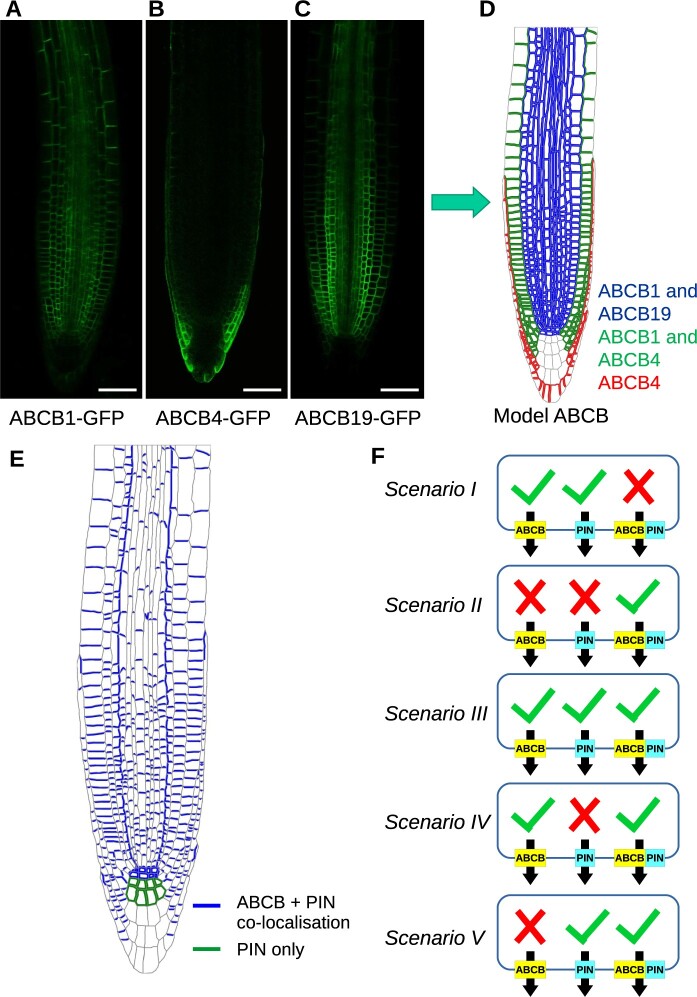
Spatial localization of the ABCB and PIN efflux transporters and summary of possible ABCB–PIN interaction scenarios. A, ABCB1–GFP. B, ABCB4–GFP. C, ABCB19–GFP. D, Spatial localization of ABCB1, ABCB4, and ABCB19 in a multicellular root-tip template. E, Spatial localization of PIN in a multicellular root-tip template. F, Five hypothetical scenarios for ABCB and PIN interaction and activity. (I) PIN and ABCB are independent. (II) PIN and ABCB are entirely co-dependent. (III) PIN and ABCB act independently but there is additional efflux when both are co-localized. (IV) ABCBs act independently, but PIN requires ABCB to act. (V) PINs act independently, but ABCB requires PIN to act.

The best-known class of auxin efflux transporters is the PIN-formed (PIN) family, which are often polarly located on specific cell membranes and create a directed auxin flux (Blilou et al., 2005; Adamowski and Friml, 2015). In addition to PINs, auxin efflux is also mediated by ATP-binding cassette transporters of the B sub-family (ABCBs) (Verrier et al., 2008; Geisler et al., 2017). In contrast to PINs, ABCBs show a reduced degree of polarity, especially toward the root tip (Figure 1, A–C; Geisler and Murphy, 2006). Furthermore, while PINs enable anionic auxin to move with the electrochemical gradient from cytoplasm to apoplast, ABCBs are primary active, ATP-driven transporters and as such can move auxin against the gradient (Geisler and Murphy, 2006). Experiments using heterologous expression in yeast and mammalian cells suggest ABCB1 and ABCB19 function as auxin efflux transporters (Geisler et al., 2005; Bouchard et al., 2006). However, studies on ABCB4 have suggested that ABCB4 can act as both an efflux transporter (Cho et al., 2007) and an influx transporter (Santelia et al., 2005; Terasaka et al., 2005, Blakeslee et al., 2007). Currently, ABCB4 is seen as a facultative im/exporter whose transport directionality depends on cellular auxin concentrations (Yang and Murphy, 2009; Kamimoto et al. 2012; Kubeš et al., 2012). Auxin import is also provided by the AUX1/LAX family of auxin influx carriers, which are mostly apolarly located within a given cell (Swarup et al., 2001, 2005).

The complexity in auxin transport activity is increased further when considering potential functional interactions between the ABCB and PIN proteins. Though a clear proof of such direct interactions has not yet been shown (Geisler et al., 2017), some synergy in activity between PINs and ABCBs is supported at both genetic (Mravec et al., 2008) and cellular levels (Blakeslee et al., 2007; Deslauriers and Spalding, 2021). More specifically, in heterologous systems, co-expression of PIN1 and either ABCB1 or ABCB19 appears to enhance auxin efflux activity, pairing PIN2 with ABCB1 and ABCB19 does not (Blakeslee et al., 2007). In addition, co-expression of ABCB4 and PIN1 appears to result in net cellular efflux, while co-expression of ABCB4 and PIN2 results in net cellular influx (Blakeslee et al., 2007). Furthermore, a recent electrophysiology study suggested that ABCB4 and PIN2 produce a synergistic efflux that is approximately double that of either protein alone (Deslauriers and Spalding, 2021). In summary, there is indication for a synergistic and antagonistic interference of ABCB–PIN-mediated auxin transport; however, due to a possible interference of PINs or ABCBs with endogenous transporter homologs/orthologs in homologous or heterologous expression systems, respectively, the individual role of ABCBs and PINs has not been dissected.

In this systems biology-based study, we analyze the role of the ABCB transporters by focusing on the auxin distribution in the Arabidopsis root tip. In root tissues, auxin regulates lateral root initiation and emergence (Péret et al., 2009), vascular patterning (Bishopp et al., 2011; De Rybel et al., 2014), root hair growth (Pitts et al., 1998; Knox et al., 2003; Jones et al., 2009), gravitropism (Bennett et al., 1996; Chen et al., 1998; Luschnig et al., 1998), and root growth rate (Blilou et al., 2005; Rahman et al., 2007). Many computational modeling studies have investigated how PIN efflux transporters and AUX1/LAX influx transporters mediate the root-tip auxin distribution (Swarup et al., 2005; Grieneisen et al., 2007; Jones et al., 2009; Band et al., 2014; Van den Berg et al., 2016; Xuan et al., 2016; Di Mambro et al., 2017). In particular, polar PIN transporters have been shown to create a directed flux in a rootward direction within the stele and in a shootward direction through the outer layers, giving rise to the so-called reverse fountain model (Grieneisen et al., 2007), while including AUX1/LAX influx transporter localization revealed PINs direct the flux through the tissue, but sites of high auxin accumulation are predominantly determined by the position of the influx carriers (Band et al., 2014). Root-tip models have also investigated additional auxin fluxes through plasmodesmata (Mellor et al., 2020), crosstalk between auxin and other hormones (Moore et al., 2015; Di Mambro et al., 2017), auxin dynamics in growing tissues (Grieneisen et al., 2007), and vasculature patterning (Muraro et al., 2013).

Detailed descriptions of ABCB-mediated auxin transport have not been included in previous computational models. Near the root tip, there are at least three ABCBs expressed: ABCB1 and ABCB19, which have strongly overlapping expression patterns in the stele, endodermis, and cortex, with ABCB1 additionally expressed in the epidermis and inner layers of the lateral root cap (LRC) (Blakeslee et al., 2007; Wu et al., 2007; Mravec et al., 2008), and ABCB4, which is restricted to the outer tissues and is found in the LRC, epidermis, and columella (Cho et al., 2007; Wu et al., 2010; Kubeš et al., 2012). An additional ABCB transporter, ABCB21, is also expressed in the pericycle in the mature root (Kamimoto et al., 2012), where it is thought to have a role in auxin homeostasis in the stele (Jenness et al., 2019). A recent study suggests that based on the conservation of a diagnostic D/E-P motif, 11 of the 22 full-size ABCBs might function as auxin transporters (Hao et al., 2020).

Experimental studies have shown that ABCBs affect root growth and development. Although single *abcb1* and *abcb19* loss-of-function mutants only showed subtle effects on primary root growth and gravitropic bending, the double mutant *abcb1 abcb19* exhibited significantly altered primary root growth, defective gravitropic bending and twisting of epidermal root files, while the *abcb4* single mutant had enhanced root gravitropism (Geisler et al., 2003; Bouchard et al., 2006; Lewis et al., 2007; Wu et al., 2007, 2010). Mutants *abcb1*, *abcb19*, and *abcb1 abcb19* (but not *abcb4*) also exhibited fewer lateral roots (Lin and Wang, 2005; Wu et al., 2007), which were shown to be caused by reduced postemergence growth (Wu et al., 2007). Furthermore, ABCB4 has been shown to regulate root hair elongation (Santelia et al., 2005; Cho et al., 2007). Experiments with radiolabeled auxin tracers revealed that ABCBs contribute to long-distance auxin transport. Reduced rootward auxin transport has been observed in *abcb1*, *abcb19*, and *abcb1 abcb19*, and shootward transport was reduced in *abcb4* (Noh et al., 2001; Geisler et al., 2003, 2005; Lewis et al., 2007). Phenotypes in double mutants between PINs and ABCBs have also provided support for ABCB–PIN synergistic interactions (Blakeslee et al., 2007; Mravec et al., 2008). Furthermore, in the *twisted dwarf 1* (*twd1*) mutant, where ABCB1, ABCB4, and ABCB19 do not reach the cell membrane (accumulating instead on the endoplasmic reticulum), primary root length and long-distance auxin transport is reduced in light-grown seedlings (Geisler et al., 2003  Wu et al., 2010). Both *twd1* and *abcb1 abcb19* exhibited twisting of the epidermal cell files in the elongation zone (EZ), assumed to be due to perturbed auxin distributions (Geisler et al., 2003, Wu et al., 2010). Taken together, these experimental studies point to an important role for ABCBs in root auxin distribution, and this is worthy of examination at a systems level.

In this study, we address the complexity presented by the overlapping expression of ABCB transporters using a systems approach to analyze their function. By explicitly modeling different hypotheses as to how ABCBs function at a subcellular scale, we predict how these hypotheses affect auxin distribution at the organ scale. We incorporate ABCBs into an existing multicellular computational model of the auxin dynamics in the root tip, which features the established localizations of PIN and AUX1/LAX transporters, and plasmodesmata (Band et al., 2014; Mellor et al., 2020; Supplemental Figure S1, D–K ). We consider several competing hypotheses to investigate potential interactions between PINs and ABCBs and evaluate how they affect the overall root tip auxin distribution and fluxes. By comparing the computational model predictions with experimentally derived auxin distributions in wild-type and single and double *abcb* mutant genotypes crossed with the DII-VENUS auxin reporter (Band et al., 2012; Brunoud et al., 2012), we find that the model can recapitulate the experimental observations only for specific ABCB–PIN regulatory interactions. These findings are further validated by simulating dynamic auxin distributions to understand why long-distance auxin transport is reduced in *abcb4* and *abcb1 abcb19* mutants. Our study thus provides insights into ABCB–PIN regulatory interactions and their role in controlling auxin patterning.

## Results

### Incorporating ABCB distributions into a computational model of the Arabidopsis root tip tissues

Using GFP-fusion marker lines for ABCB1, ABCB4, and ABCB19, we observed that, as previously reported (Cho et al., 2007; Blakeslee et al., 2007; Wu et al., 2007; Mravec et al., 2008; Wu et al., 2010; Kubeš et al., 2012), ABCB1 is widely expressed in the root except for the outer LRC and columella, ABCB19 is expressed within the stele and pericycle, endodermis and cortex, and ABCB4 is restricted to the outer LRC, the epidermis and the outermost tier of the columella (Figure 1, A–C, see Supplemental Figure S2 for cell types). The ABCBs appear to have a widely nonpolar distribution within the cells in this region, as observed by Geisler et al. (2005), Cho et al. (2007), Wu et al. (2007), Mravec et al. (2008), and Wu et al. (2010). Using these observations, we formulated rules for the ABCB distributions (Figure 1D; Supplemental Figure S1, A–C). In most tissues, two ABCBs are present, while in the epidermis and outer LRC only ABCB4 is present, and in the inner tiers of the columella, these ABCBs are not present.

We incorporated the ABCB distributions (Figure 1D) into an established vertex-based model of the auxin dynamics within the Arabidopsis root tip (Band et al., 2014; Mellor et al., 2016; Xuan et al., 2016; Mellor et al., 2020). This model simulates auxin dynamics within real multicellular root tip geometries that are segmented from confocal images of root tips stained with propidium iodide, using the SurfaceProject and CellSeT image analysis tools (Pound et al., 2012; Band et al., 2014). Distributions of PIN, AUX1/LAX, ABCB, and plasmodesmata were automatically specified on these root-tip templates, with the PIN and AUX1/LAX distributions based on the antibody and YPet reporter data by Band et al. (2014) (see Figures 1E; Supplemental Figure S1, D–K), and the plasmodesma distribution following the electron microscopy data of Zhu et al. (1998) (as in Mellor et al., 2020). Using the multicellular root-tip template and membrane–protein distributions, the model uses a system of ordinary differential equations (ODEs) to describe passive diffusion of protonated auxin across cell membranes, active transport of anionic auxin across cell membranes via PIN, AUX1/LAX, and ABCBs, cell-to-cell auxin diffusion through plasmodesmata, passive auxin diffusion within the cell wall, and auxin synthesis and degradation (see “Materials and Methods” and “Supplemental Methods” for further details).

### Modeling competing scenarios for regulatory interactions between ABCB and PIN proteins

Experimental studies have suggested that PIN and ABCB proteins interact to mediate auxin transport (Blakeslee et al., 2007; Mravec et al., 2008), leading to various hypotheses as to what form these interactions might take. Following the suggestions in the reviews of Spalding (2013) and Geisler et al. (2017), we considered five possible scenarios, as summarized in Figure 1F:

1. Both ABCB and PIN are independent, and neither the presence nor absence of the other affects their auxin efflux.
2. The ABCB and PIN proteins are entirely co-dependent, and both must be present on a given membrane for either to efflux auxin.
3. ABCB and PIN act independently, but where both are present on a given membrane there is an additional synergistic flux increasing the total rate of efflux.
4. ABCB proteins efflux auxin independently; however, PINs are not able to transport auxin in the absence of ABCBs but instead enable a co-dependent efflux where both ABCB and PIN are present.
5. PIN proteins efflux auxin independently; however, ABCBs are not able to transport auxin in the absence of PINs but instead enable a co-dependent efflux where both ABCB and PIN are present.

These five scenarios are implemented in the model by considering the transporter-mediated auxin efflux at each membrane segment to be the sum of three possible efflux components: an ABCB-mediated flux, which is proportional to the number of ABCB family members present, a PIN-mediated flux, which is proportional to the number of PIN family members present, and a synergistic or co-dependent flux, which is proportional to the both the number of PIN family members and the number of ABCB family members present (see “Supplemental Methods” for further details). To simulate each scenario, a different combination of these fluxes can be set as shown in Figure 1F. We supposed initially that, when present, the permeabilities associated with each PIN and ABCB family member and with the co-dependent efflux are equal (see Eqs. (1) and (2) in the “Supplemental Methods”) and present results demonstrating how our conclusions are affected by changing these parameter values (as detailed below). Scenario III is supported by results from heterologous expression systems, which have suggested both independent and interactive ABCB and PIN transport (Bouchard et al., 2006; Blakeslee et al., 2007; Geisler et al., 2017; Deslauriers and Spalding, 2021). However, these heterologous results may be affected by the presence of endogenous transporters in such systems, therefore, motivating further examination of these scenarios *in planta* using a systems approach. We note that in Scenario IV, for example, in which PINs transport auxin only in the presence of ABCBs, the co-dependent flux could represent two possible situations, either (1) the PINs are not auxin transporters *per se* but instead increase ABCB-mediated efflux (where both PIN and ABCB are present) or (2) the PINs are auxin transporters, but require activation by ABCBs for auxin efflux. Our model (and results) is unable to distinguish between these two possibilities (see “Discussion” for further details).

### ABCB–PIN regulatory interactions influence predicted root-tip auxin distribution

**Figure 2:**
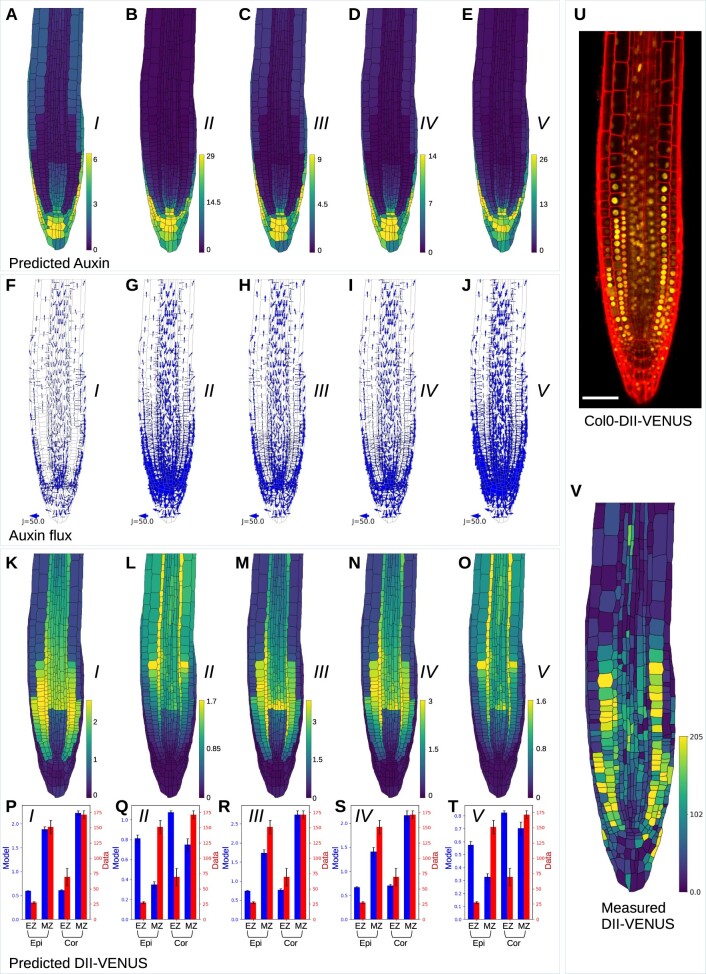
Wild-type model predictions for each of the five ABCB–PIN interaction scenarios and experimental data showing the root-tip DII-VENUS distribution. A–E, Predicted auxin concentrations for each of the five ABCB–PIN interaction scenarios. F–J, Predicted auxin fluxes between cells for each of the five ABCB–PIN interaction scenarios. K–O, Predicted DII-VENUS concentrations for each of the five ABCB–PIN interaction scenarios. P–T, Mean DII-VENUS in the meristematic and EZ regions of the epidermis and cortex, comparing the model predictions for each of the five ABCB–PIN interaction scenarios (shown in K–O) with the experimental data (shown in V). Error bars show ±1 se. U, Confocal image of a Col0-DII-VENUS root tip, showing DII-VENUS (yellow) and cell geometries (red) (via propidium iodide staining). V, Measured DII-VENUS levels extracted from the image in (U).

To investigate whether the ABCB–PIN regulatory interactions affect the root-tip auxin distribution, we simulated the model in a real multicellular root-tip template segmented from an image of a wild-type seedling. Simulating the wild-type model for each of the five ABCB–PIN interaction scenarios in turn, the model predicted qualitatively similar auxin distributions in all five scenarios, with a reversed fountain flux pattern and high auxin levels close to the quiescent center (Figure 2, A–J).

The choice of ABCB–PIN interaction, however, affects auxin accumulation in the epidermis and cortex of the EZ, with Scenario I showing the greatest auxin accumulation (Figure 2A), Scenarios III and IV showing lower auxin accumulation (Figure 2, C and D), and Scenarios II and V having no noticeable auxin accumulation (Figure 2, B and E). Thus, epidermal and cortical auxin accumulation is predicted to only occur if the ABCBs efflux auxin independently (Scenarios I, III, and IV), which enables lateral ABCB-mediated efflux into the apoplast between adjacent cell tissue layers. These predictions were further validated by simulating the auxin distributions in a geometrically regular template (similar to that used by Van den Berg et al. (2016) and Di Mambro et al. (2017)), which were determined to be consistent with those using the real multicellular geometries (see “Supplemental Methods”; Supplemental Figures S2 and S3; Supplementary Table S1).

The root-tip auxin flux pattern is essential to many developmental processes, including gravitropism and lateral root initiation, whereby directed auxin fluxes enable cells to undergo a coordinated response. Although visualizing auxin flux patterns experimentally is challenging, a modeling approach enables us to predict the flux pattern and to assess how this pattern is affected by the transporter properties and distributions. The model predicted that the magnitude of the directional auxin fluxes depends on the ABCB–PIN interaction scenario (Figure 2, F–J), with the smallest fluxes occurring in the case in which there is no synergistic interaction between PIN and ABCB (Scenario I, Figure 2F), and the largest fluxes in the cases where ABCB requires PIN in order to mediate efflux (Scenarios II and V; Figure 2, G and J).

We conclude that the choice of ABCB–PIN interaction affects the predicted wild-type auxin distribution and flux pattern. Comparing the model predictions with experimentally derived distributions may enable us to suggest which interaction is occurring.

### Experimentally validated model predictions reveal ABCBs act independently of PINs

To validate our model experimentally, we used the nucleus-localized yellow fluorescent protein (YFP) auxin reporter DII-VENUS (which is an Aux/IAA-based reporter composed of a constitutively expressed fusion of the auxin-binding domain (DII) of the Aux/IAA28 protein to a fast-maturating variant of YFP, VENUS; Brunoud et al., 2012). Auxin rapidly degrades DII-VENUS through a small protein interaction network and thus imaging the root-tip DII-VENUS distribution provides an accurate readout of the auxin distribution (with high DII-VENUS corresponding to low auxin and vice versa).

We previously developed and parameterized a mechanistic model of the network of interactions through which auxin promotes DII-VENUS degradation (Band et al., 2012) (see “Supplemental Methods” for details); by simulating this network model in every cell, we can use this parameterized network model to predict the DII-VENUS distribution within the root tip (Band et al., 2014). By comparing the observed and predicted DII-VENUS distributions, we can assess which of the five ABCB–PIN interaction scenarios can recapitulate the experimental data.

We simulated the model in a multicellular root-tip template segmented from an image of a wild-type seedling crossed with the DII-VENUS auxin reporter, and quantitatively compared the predicted and observed DII-VENUS, focusing on the epidermis and cortex, as these tissues have the greatest resolution for both the cell walls and nuclei in the experimental images (Figure 2, K–V). These tissue layers are then further sub-divided into EZ and meristematic zone (MZ), and we calculated the mean (and standard error) DII-VENUS for both model and data in each subset of cells. Predictions with Scenarios II and V show poor agreement with the experimental data, with the model predicting high DII-VENUS in the EZ epidermis and low DII-VENUS in the MZ epidermis, and vice versa in the experimental data (Figure 2, L, O, Q, and T). Furthermore, predicted DII-VENUS levels in Scenarios II and V (Figure 2, L and O) are much higher in the EZ all across the root than is seen experimentally. Predictions with Scenarios I, III, and IV, however, all show reasonable agreement with the data (Figure 2, K, M, N, P, R, and S). Quantitatively comparing the experimental data with model predictions for a wide range of permeability parameters showed that these findings are robust against changes in these parameters (Supplemental Figure S4A).

In summary, comparison of the model predictions with experimental data in wild-type suggests that Scenarios II and V are unlikely to be correct. In the remaining scenarios, the nonpolar ABCBs enable auxin to efflux through the periclinal cell membranes, entering the apoplast between the adjacent tissues, where AUX1 mediates influx into specific tissues. Thus, our results suggest that the investigated ABCBs are able to act independently of PINs.

### Model validation using *aux1* and *pin2* mutants confirms ABCBs efflux independently of PINs

To further investigate the ABCB–PIN interactions, we simulated the auxin and DII-VENUS distributions in *aux1* and *pin2* mutants (Supplemental Figures S5 and S6) (which we previously modeled by Band et al. (2014) and Mellor et al. (2020) respectively). Auxin predictions for *aux1* show similar patterns for all five ABCB–PIN scenarios, with high auxin maximum in the QC region and low auxin throughout the EZ (Supplemental Figure S5, C–L), similar to those produced with previous versions of the model (Band et al., 2014). The corresponding DII-VENUS predictions (Supplemental Figure S5, M–Q) are in reasonable agreement with the experimental observations (Supplemental Figure S5, B, R–V).

For *pin2*, we see differences between the predicted auxin and DII-VENUS distributions for Scenarios I, III, and IV and Scenarios II and V (Supplemental Figure S6, C–Q), as in the wild-type predictions (Figure 2, A–O). For Scenarios I, III, and IV, the model predicted high auxin levels and fluxes in the LRC and EZ epidermis and cortex, whereas for Scenarios II and V, the predicted auxin levels and fluxes are lower in these outer tissues (Supplemental Figure S6, C–L). Thus, the model predicted high auxin levels in the outer layers, in agreement with the experimental data (Supplemental Figure S6, A and B), only if ABCBs are able to independently efflux auxin (Scenarios I, III, and IV). We note, however, that the model predicted lower DII-VENUS in the EZ than in the MZ in these layers, which is not apparent in the experimental data.

We conclude that the *aux1* predictions agree with the data for any of the five scenarios, whereas for *pin2* we only obtain agreement between the predictions and data for Scenarios I, III, and IV. Thus, the *pin2* predictions provide further evidence that ABCB efflux auxin independently of PIN.

### The model predicts that ABCB4 does not mediate auxin influx in the root tip

In the simulations above, we assumed that all three ABCBs efflux auxin equally and only differ in spatial localizations. However, some experimental studies suggest ABCB4 acts as an influx transporter, at least at low cytoplasmic auxin concentrations (Santelia et al., 2005; Kamimoto et al., 2012). To investigate this possibility, we used the model to predict the auxin and DII-VENUS distributions supposing that ABCB4 acts as an influx transporter at the auxin concentrations present at the root tip (with equal weight as the influx carriers AUX1, LAX2, and LAX3). Comparing wild-type predictions with ABCB4 acting as an influx transporter and an efflux transporter (Supplemental Figure S7, A–E; Figure 2, K–O), we see that the key difference occurs in the meristematic epidermis which is the only tissue where ABCB4 does not coincide with the AUX1 influx carrier. As one might expect, introducing an ABCB4-mediated influx into the meristematic epidermis leads to higher auxin levels (and lower DII-VENUS) in this tissue (Supplemental Figure S7, A–E), which is not in agreement with the experimental DII-VENUS data (Figure 2, U and V).

It is possible that in wild-type any influx activity of ABCB4 may be masked by the presence of AUX1. To test this hypothesis, we simulated the auxin dynamics in an *aux1* mutant with ABCB4 operating as an influx transporter. The model predicted low DII-VENUS (high auxin) in the meristematic epidermis and LRC in every case (Supplemental Figure S7, F–J), and low DII-VENUS in the EZ epidermis in Scenarios I, III, and IV (Supplemental Figure S7, F, H, and I). These predictions do not agree with the *aux1* DII-VENUS images (Supplemental Figure S5A), which show high DII-VENUS throughout the root tip except in the QC region.

These model predictions with ABCB4 mediating auxin influx do not agree with DII-VENUS images in both wild-type and *aux1*, and thus, our results suggest that ABCB4 does not function as an influx transporter in the root tip. Given auxin levels in the root tip are relatively high, our finding is consistent with the suggestions that ABCB4 mediates influx only at low auxin concentrations (Yang and Murphy, 2009; Kamimoto et al., 2012; Kubeš et al., 2012). For this reason, from this point on we assume that ABCB4 is operating as an auxin efflux transporter in the root tip with equal activity (when present) as ABCB1 and ABCB19.

### ABCB–PIN interactions affect the predicted DII-VENUS for the *abcb1 abcb19* mutant

For wild-type and *pin2*, the model predictions and experimental data are in reasonable agreement provided ABCBs mediate auxin efflux independently of PINs (Figure 2; Supplemental Figure S6) (Scenarios I, III, and IV). We hypothesized that the effects of the differences between these Scenarios I, III, and IV may become more obvious when individual ABCBs are deleted.

**Figure 3:**
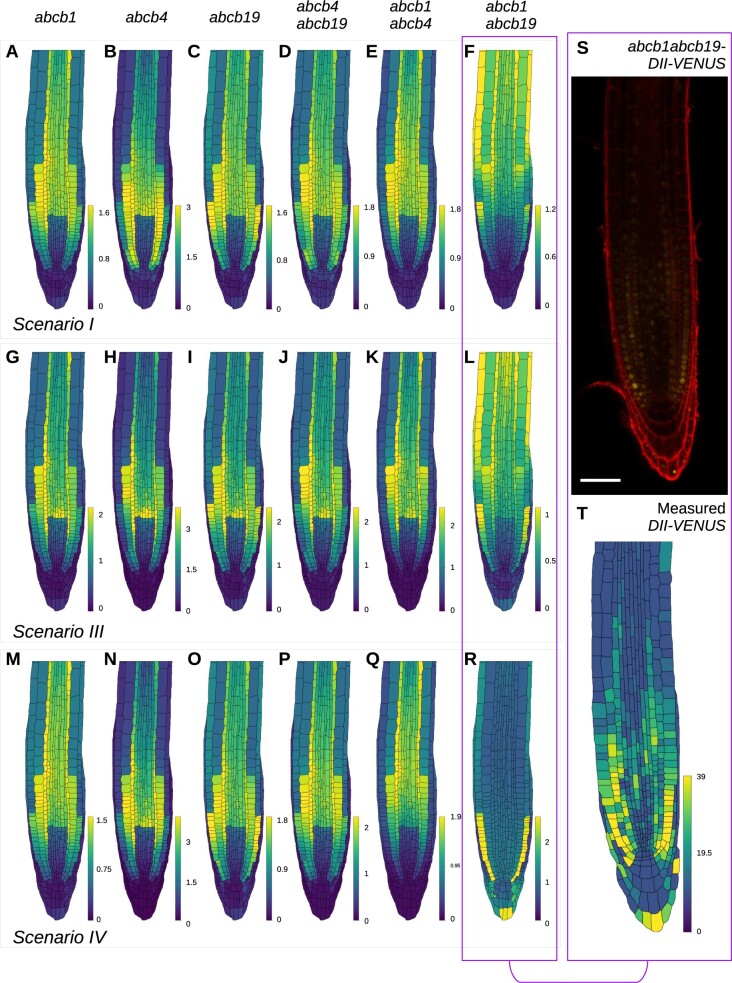
Predicted DII-VENUS distributions for *abcb* single and double loss-of-function mutants for Scenarios I, III, and IV, together with experimental data showing the DII-VENUS distribution in *abcb1 abcb19*. A–R, Predicted DII-VENUS distribution for *abcb1* (A, G, and M), *abcb4* (B, H, and N), *abcb19* (C, I, and O), *abcb4 abcb19* (D, J, and P), *abcb1 abcb4* (E, K, and Q), and *abcb1 abcb19* (F, L, and R), in each of the three favored Scenarios I (A–F), III (G–L), and IV (M–R). S, Confocal image of *abcb1 abcb19*-DII-VENUS root-tip showing DII-VENUS (yellow) and cell geometries (red) (via propidium iodide staining). T, Measured DII-VENUS levels extracted from the image in (S).

Focusing our attention on the remaining Scenarios I, III, and IV, we used the model in the wild-type template to predict auxin and DII-VENUS distributions for the single mutants *abcb1*, *abcb4*, *abcb19*, and the double mutants *abcb4 abcb19, abcb1 abcb4, abcb1 abcb19* under Scenario I (Figure 3, A–F), Scenario III (Figure 3, G–L), and Scenario IV (Figure 3, M–R). In each case, the mutants were simulated by setting the value of the relevant ABCB transporter(s) to zero throughout the root tip. In most mutants, while there are some differences, the overall predicted auxin and DII-VENUS patterns are qualitatively similar to wild-type for all three scenarios, with high auxin and low DII-VENUS levels in the QC region, LRC and EZ epidermis and cortex. Notably, however, we observed that one mutant has not only different predicted DII-VENUS pattern from the other genotypes, but also different predicted patterns between the different scenarios, that mutant being *abcb1 abcb19*. In *abcb1 abcb19*, for Scenarios I and III, the model predicted an auxin maximum in the QC region, with low auxin and high DII-VENUS throughout the remainder of the root tip (Figure 3, F and L), whereas for Scenario IV, the model predicted an unusual pattern with low auxin and high DII-VENUS in only the meristematic epidermis (Figure 3R).

In Scenario IV, PIN does not efflux independently of ABCB (in contrast to Scenarios I and III); therefore, in the *abcb1 abcb19* double mutant, PIN efflux operates only where ABCB4 is present (i.e. in the LRC and epidermis). Thus, for Scenario IV, in *abcb1 abcb19* both the ABCB-mediated nonpolar efflux and the rootward PIN-mediated directed flux does not operate in the stele, pericycle, and endodermis; auxin that enters the root tip from the shoot/phloem is not advected toward the QC and instead diffuses between adjacent cells through plasmodesmata resulting in the predicted uniform auxin distribution within the EZ. In the meristem, ABCB-mediated efflux operating in the epidermis and LRC, together with AUX1-mediated influx in the LRC, results in the model predicting low auxin levels (high DII-VENUS) in the meristematic epidermis (Figure 3R).

Given that the ABCB–PIN interaction scenario affects the predicted *abcb1 abcb19* auxin distribution, we concluded that the *abcb1 abcb19* mutant warranted further study. We reasoned that experimentally observing the DII-VENUS distribution in *abcb1 abcb19* and comparing the observed distribution with the model predictions may suggest which ABCB–PIN interaction scenario is acting in the root tip.

### Validated model reveals that PINs and ABCBs mediate a co-dependent efflux

Since the model (in the wild-type template) indicated that the choice of ABCB–PIN interaction affected the predicted DII-VENUS pattern in the *abcb1 abcb19* mutant, we focused on this line, crossing DII-VENUS into an *abcb1 abcb19* mutant background (Figure 3S, see Supplemental Figure S8 for replicates). Previous studies have revealed auxin-related phenotypes in *abcb1 abcb19*, including defective primary root growth, gravitropic bending and root twisting, and fewer lateral roots (Geisler et al., 2003; Lin and Wang, 2005; Bouchard et al., 2006; Wu et al., 2010). However, studies using the DR5 reporter in *abcb1 abcb19* have suggested both high and low auxin levels in the columella region (Geisler et al., 2005; Bouchard et al., 2006; Mravec et al., 2008; Wu et al., 2010), further motivating our investigation of *abcb1 abcb19* using the DII-VENUS reporter.

We observed overall reduced DII-VENUS (compared to wild-type), corresponding to high auxin levels, which is consistent with the model predictions (Figure 3S). Quantifying the nuclear intensities of DII-VENUS in *abcb1 abcb19* revealed that DII-VENUS levels are relatively high underlying the LRC, especially in the epidermis, and are low and uniform throughout the EZ (Figure 3T). Qualitatively comparing the model predictions and experimental data for *abcb1 abcb19* (Figure 3, F, L, R, and T) reveals that the predicted DII-VENUS distributions under Scenario IV is in reasonable agreement with the DII-VENUS data, whereas predictions for Scenarios I and III are substantially different.

To test whether these conclusions are robust to changes in model parameter values, we compared quantitatively the *abcb1 abcb19* experimental data and model predictions using a range of permeability parameters for Scenarios I, III, and IV (Supplemental Figure S4B). Interestingly, for Scenario III as we increased the magnitude of the co-dependent efflux, the agreement between the predictions and data increased. Thus, although Scenario IV predictions agreed best with the data for the majority of parameter sets, if the co-dependent efflux is large (at least five times larger than the PIN and ABCB efflux alone) then Scenario III recapitulates the data most accurately.

We conclude that the model predictions and data suggest that PINs and ABCBs contribute to a co-dependent auxin efflux where both are present. Our results suggest that PINs either only efflux auxin through this co-dependent efflux (Scenario IV) or efflux auxin independently of ABCBs but at a much lower rate than the co-dependent efflux (Scenario III, with co-dependent permeability at least five times larger than those of the PINs and ABCBs alone).

### Validated model predicts how ABCBs affect root-tip auxin distribution

**Figure 4:**
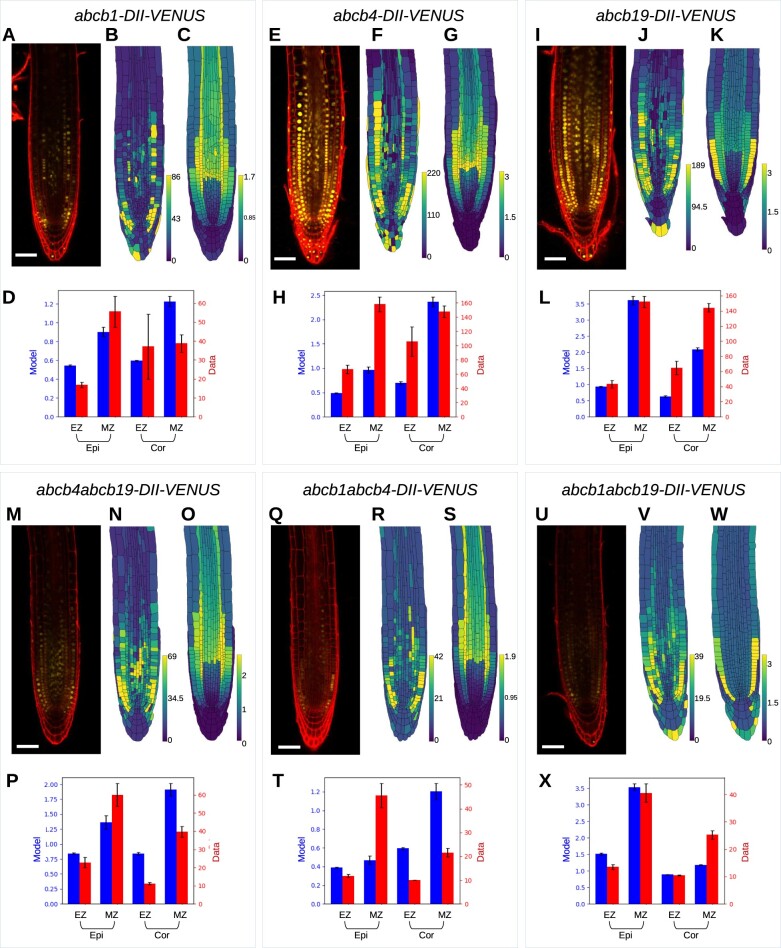
Model predictions and experimental data for *abcb* single and double loss-of-function mutants. The model predictions use Scenario IV and integrate RT-qPCR data which quantifies the small upregulation of the remaining ABCBs in the mutant lines. (A–D) *abcb1*, (E–H) *abcb4*, (I–L) *abcb19*, (M–P) *abcb4 abcb19*, (Q–T) *abcb1 abcb4*, and (U–X) *abcb1 abcb19*. A, E, I, M, Q, and U, Confocal images showing DII-VENUS (yellow) with propidium iodide background staining (red). B, G, J, N, R, and V, Measured DII-VENUS levels extracted from the corresponding confocal image. C, G, K, O, S, and W, Predicted DII-VENUS distributions. D, H, L, P, T, and X, Mean DII-VENUS in the meristematic and EZ regions of the epidermis and cortex, comparing the model predictions and experimental data. Error bars show ±1 SE (standard error). We note that to aid readability, the data presented in Figure 3, S and T are repeated here in Figure 4, U and V.

To test the model further, we created new lines by crossing the *abcb1*, *abcb4*, *abcb19*, mutant lines with the DII-VENUS reporter lines and then used those to generate double mutant DII-VENUS reporter lines for *abcb1 abcb4* and *abcb4 abcb19* (Figure 4, A, E, I, M, and Q, see Supplemental Figures S8 and S9 for replicates). Using images from these lines to create root-tip templates for each mutant and removing the corresponding ABCBs from our simulations, we predicted the auxin and DII-VENUS distributions for each mutant in the relevant root-tip template. Comparing the predicted DII-VENUS patterns with those observed, we found reasonable agreement between predictions and data in *abcb19*, *abcb4 abcb19*, and *abcb1 abcb19*, although some differences for *abcb1, abcb4*, and *abcb1 abcb4* (Supplemental Figure S10). We hypothesized that differences could be caused by compensatory upregulation of ABCB19 (previously suggested for *abcb1* in Jenness et al. (2019)). To test this hypothesis, we quantified the expression of each ABCB in each of the mutant backgrounds (Supplemental Figure S11). We observed only a small upregulation of the remaining ABCBs for most of the mutant lines, with the exception of *ABCB19* expression in the *abcb1 abcb4* mutant, which was increased 2.3-fold. Integrating these data into the model resulted in only small modifications to the predicted distributions (compare Figure 4 and Supplemental Figure S10). Furthermore, we observed that whether PINs contribute only to the co-dependent efflux (Scenario IV, Figure 4) or also mediate a smaller independent efflux (Scenario III, Supplemental Figure S12) makes only small differences to the predicted distributions for these mutants.

In the three single mutants, *abcb1*, *abcb4*, and *abcb19*, we observed a DII-VENUS distribution that is similar to wild-type (compare Figure 4, A–L with Figure 2, N, S, and V). In the *abcb1* mutant, we see much weaker overall DII-VENUS (corresponding to higher auxin) in both the data and the model predictions (Figure 4, A–D) (leading to more patchy expression levels in the data, as the fluorescence in some cells falls below the detection threshold). Through the synergistic efflux, ABCB1 contributes to the shootward transport in the epidermis, explaining the higher auxin (lower DII-VENUS) in the *abcb1* results.

**Figure 5:**
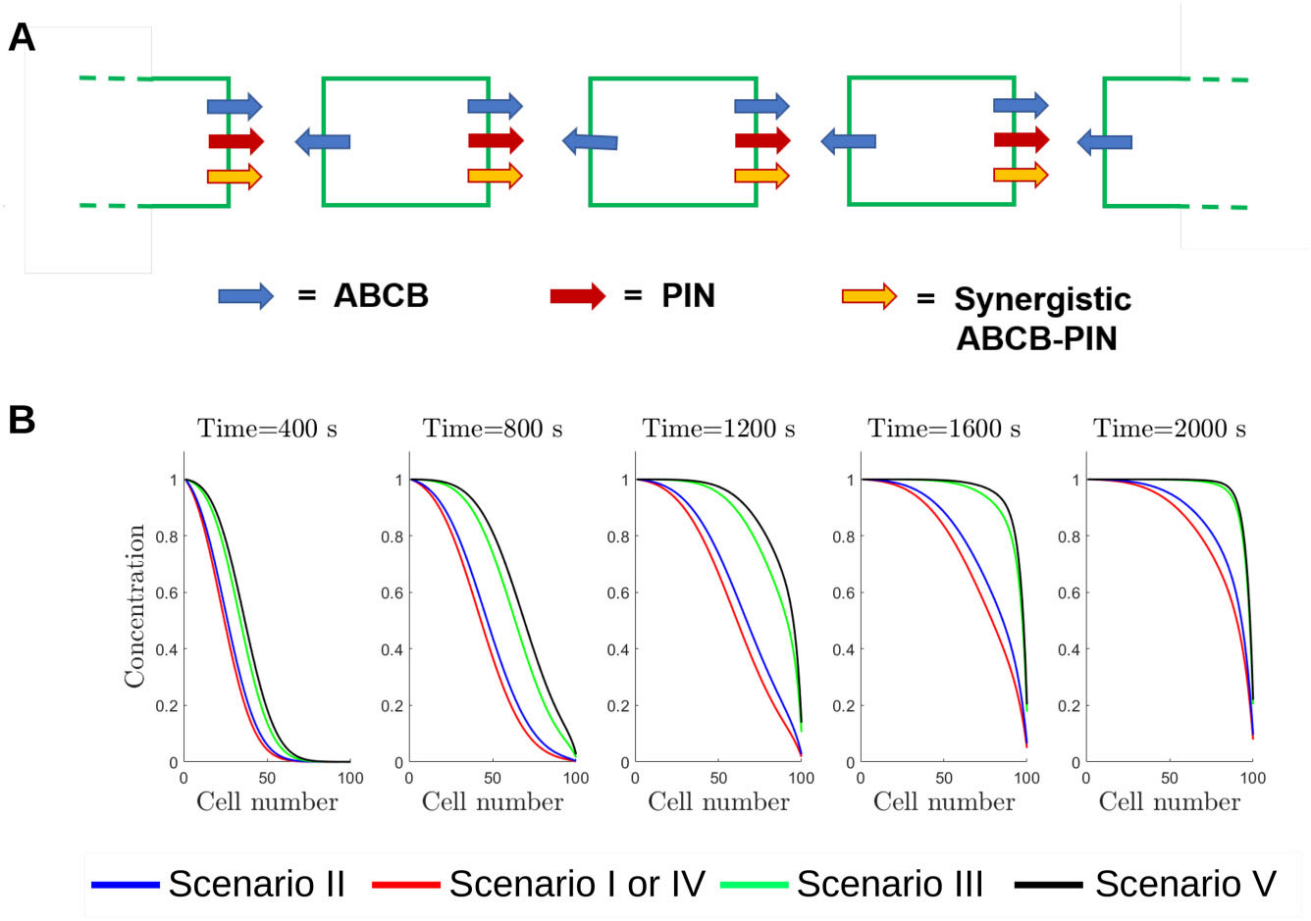
Effect of PIN and ABCB-mediated efflux on the auxin propagation through a single-cell file. A, Schematic showing the distribution of the PIN, ABCB, and synergistic auxin efflux components. B, Predicted auxin distribution at different times for the five ABCB–PIN interaction scenarios. We supposed the auxin concentration in cell 0 is fixed to be equal to 1, and all other cellular concentrations are zero at time *t*  =  0. Including the synergistic efflux due to ABCB and PIN co-expression (PIN–ABCB) increases the speed of the auxin wave, while including efflux due to apolar ABCB marginally decreases the speed of auxin transport.

The predicted DII-VENUS distribution in *abcb4* is also similar to wild-type (Figure 4, E–H), although with lower DII-VENUS (higher auxin) in the meristematic epidermis, where the ABCB-mediated and synergistic efflux are reduced (compare Figures 2, S and 4, H). It is unclear why this lower DII-VENUS in these tissues is not seen in the *abcb4* data (which show similar DII-VENUS levels to wild-type), one possible explanation being the presence of other, as yet uncharacterized, members of the ABCB family.

The DII-VENUS distribution for *abcb19* is very similar to wild-type in both the predictions and experimental data (Figure 4, I–L), as one may expect given ABCB1 is localized in the same tissues as ABCB19. Both the *abcb19* predictions and data suggest this leads to higher DII-VENUS in epidermis than cortex in the meristem (Figure 4, K and L), in contrast to wild-type (Figure 2, N and S) (although we note that these differences are only small in the data). This finding that *abcb19* has a similar auxin distribution to wild-type is consistent with phenotyping studies which have found no or subtle effects on primary root growth and gravitropic bending in *abcb19* (Geisler et al., 2003; Lewis et al., 2007).

We see reduced DII-VENUS levels (high auxin) in all three double mutant lines, *abcb1 abcb19*, *abcb4 abcb19*, and *abcb1 abcb4* in both the predictions and experimental data (Figure 4, M–X). For *abcb4 abcb19*, we see good qualitative agreement between model and data, with distributions that are similar to wild-type (Figure 4, M–P). The agreement between the predicted and observed DII-VENUS distributions in *abcb1 abcb4* is less clear (Figure 4, Q–T), with high DII-VENUS in the meristematic epidermis in the data, which is absent in the model. As for *abcb4*, we envisage this difference may be caused by the presence of additional ABCBs.

We also used the model to investigate how ABCB-mediated efflux affects the apoplastic auxin concentrations, since it was previously suggested that they play a role in the balance between cellular and apoplastic auxin levels (Geisler and Murphy, 2006). Considering the predicted apoplastic auxin concentration (in Scenario IV) in the single and double *abcb* mutants revealed that reducing ABCB-mediated efflux reduced the apoplastic auxin levels, especially in the *abcb1 abcb19* double mutant (Supplemental Figure S13).

In summary, with the exception of *abcb1 abcb4*, we see reasonable agreement between the model predictions and DII-VENUS data for the *abcb* single and double mutants.

### ABCB–PIN synergistic interactions enhance directed auxin flux

To further understand the role of the co-dependent ABCB–PIN efflux, we investigated the auxin flux by considering a simple model consisting of a single line of cells. We supposed that PINs are polarized to the right-hand end of each cell, that ABCBs are present at both ends and that neighboring cells are separated by an apoplastic space (Figure 5A). We supposed there to be a fixed concentration of auxin at one end of the cell file and simulated the propagation of auxin along the cell file over time (see “Supplemental Methods” for the model equations). In this simple set up, the presence of a polar efflux component leads to a directed auxin flux that can be quantified by an auxin wave speed (i.e. the speed of the propagating auxin front (Kramer et al., 2011)); however, nonpolar efflux contributes a diffusive flux that retards the auxin wave speed (Mitchison, 1980). Thus, with a polar efflux either via a PIN-mediated or a synergistic component, the model with nonpolar ABCB-mediated efflux (red lines in Figure 5B, analogous to Scenario I or IV, respectively) has a marginally reduced auxin wave speed compared to a model without the nonpolar ABCB efflux (blue lines, Scenario II). However, the auxin wave speed increases with a larger polar efflux component (Supplemental Figure S14), and, therefore, we see a large auxin wave speed if both PIN-mediated and synergistic polar fluxes are included (green lines, Scenario III). In contrast, the wave speed also increases if we decrease the magnitude of the nonpolar ABCB-mediated efflux (Supplemental Figure S14). We, therefore, see the largest wave speed occurring where the nonpolar ABCB-mediated flux is zero (black lines, Scenario V).

Taken together, these results from the single-file model demonstrate that the magnitude of the polar efflux due to both the PIN and synergistic fluxes is the key factor determining the auxin wave speed. The presence of nonpolar ABCB-mediated efflux reduces the propagation of auxin through the tissue.

### Co-dependent ABCB–PIN-mediated efflux is essential to predict the observed long-distance auxin transport

**Figure 6:**
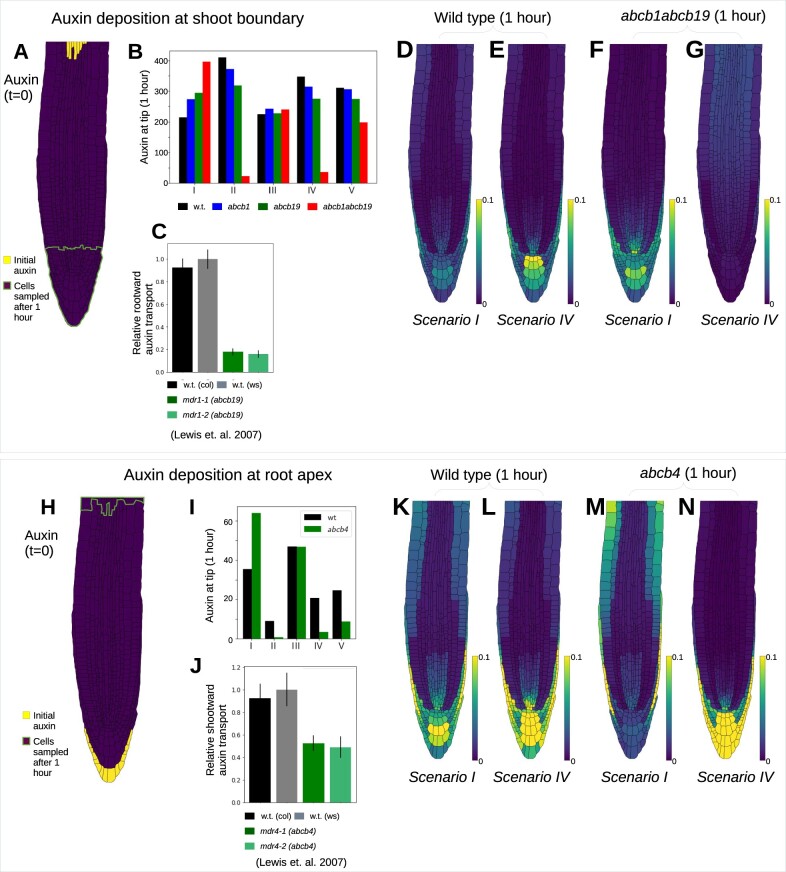
Model predictions from simulations of long-distance auxin transport. A–G, Rootward auxin transport, with auxin deposition at the shoot boundary. H–N, Shootward auxin transport, with auxin deposition at the root tip. A and H, Model initial conditions and region of cells used in (B and I) to quantify auxin after 1 h. B and I, Total predicted auxin at the root apex (B) or in the EZ (I) 1 h after auxin deposition, for each of the five ABCB–PIN interaction scenarios. C, Data reproduced from Lewis et al. (2007) showing acropetal (rootward) auxin transport measured by applying 3H-IAA to the root–shoot junction zone and later determining the amount of radioactivity in an apical portion of the root. Values shown are mean ± se of five independent trials, each involving eight roots per genotype. D and E, Predicted wild-type auxin distribution 1 h after auxin deposition at the shoot boundary for Scenarios I (D) and IV (E). F and G, Predicted auxin distribution in *abcb1 abcb19* 1 h after auxin deposition at the shoot boundary for Scenarios I (F) and IV (G). J, Data reproduced from Lewis et al. (2007) showing basipetal (shootward) auxin transport measured by applying 3H-IAA to the root apex and later determining the amount of radioactivity in a basal segment of the root. Values shown are mean ± se of seven independent trials, each involving eight roots per genotype. K and L, Predicted wild-type auxin distribution 1 h after auxin deposition at the root tip for Scenarios I (K) and IV (L). M and N, Predicted auxin distribution in *abcb4* 1 h after auxin deposition at the root tip for Scenarios I (M) and IV (N).

Previous studies have suggested ABCBs contribute to long-distance auxin transport, in both shootward and rootward directions (Noh et al., 2001; Geisler et al., 2003, 2005; Lewis et al., 2007). To further investigate the role of ABCBs using the model, we approximated previous long-distance auxin-transport experiments by initially depositing a fixed amount of auxin in a small set of cells at either the shootward boundary (Figure 6A) or at the root apex (Figure 6H) and simulating the auxin dynamics over the subsequent hour. The mean auxin per cell for each genotype and scenario is taken for a subset of cells at the apex (for the case of deposition at the boundary) or near the shootward boundary (for the case of deposition at the root apex). Motivated by the findings from the steady-state distributions, we performed simulations both with Scenario IV, with the ABCB-independent and ABCB–PIN co-dependent efflux having equal permeabilities (Figure 6), and with scenario III, with a large co-dependent permeability and small permeabilities for ABCB- and PIN-independent efflux (Supplemental Figure S15).

Considering rootward transport, Lewis et al. (2007) observed a reduction in long-distance transport in *abcb19* in the root (Figure 6C). Simulation results with both parameter sets (Figures 6B; Supplemental Figure S15A) show that the model predictions with Scenario I are clearly inconsistent with these data, whereas including a co-dependent ABCB–PIN-mediated efflux can result in model predictions that agree with the observations (Figure 6C). With the presence of a co-dependent efflux, the ABCBs contribute to the polar efflux in the stele cells; as a result, there is a reduction in polar efflux in the stele in *abcb1*, *abcb19*, and *abcb1 abcb19*, which explains the reduction in the overall rootward transport.

Visualizing the auxin dynamics after deposition at the shootward boundary further supports the finding that the co-dependent ABCB–PIN efflux is essential for ABCBs to affect long-distance rootward transport (Figure 6, D–G; Supplemental Movies S1–S4). In wild-type, there is a rapid rootward auxin flux through the stele as well as lateral diffusion via plasmodesmata; the model predicts that the deposited auxin is redistributed into a typical wild-type pattern within 15 min (Figure 6, D and E; Supplemental Movies S1 and S2). However, with ABCB removed from the stele via the *abcb1 abcb19* mutant, the auxin dynamics depend on the ABCB–PIN interaction scenario: in Scenario I, the polar efflux due to PINs remains, transporting auxin to the root tip; however, in Scenario IV, the polar efflux in the stele is disrupted in *abcb1 abcb19*, greatly reducing rootward transport (Figure 6, F and G; Supplemental Movies S3 and S4).

Considering shootward transport, Lewis et al. (2007) observed a reduction in auxin transport in *abcb4* in the root (Figure 6J); the model predictions are consistent with these data for Scenarios II, IV, and V for equal permeabilities (Figure 6I) and for II, III, IV, and V with a high co-dependent ABCB–PIN permeability (Supplemental Figure S15H). Thus, including a co-dependent ABCB–PIN-mediated efflux is again essential for model predictions to agree with observations: with a co-dependent efflux the polar efflux in the LRC and epidermal cells is reduced in *abcb4*, explaining the reduction in shootward transport. Visualizing the dynamics of the deposited auxin for scenarios I and IV, we see a substantial shootward flux in wild-type, with the deposited auxin redistributing into a typical “wild-type” pattern within 10 min (Figure 6, K and L; Supplemental Movies S5 and S6). In contrast, considering *abcb4* with Scenario IV for example, the deposited auxin does not appear to reach the EZ, as one may expect for a mutant with reduced shootward transport (Figure 6, M and N; Supplemental Movies S7 and S8).

The predictions are consistent with the results from the single-cell file simulations (Figure 5) that the polar efflux from each cell determines the long-distance auxin transport. Thus, the presence of a synergistic co-dependent efflux (enabling ABCB to contribute to the polar efflux) is essential for the ABCBs to have significant influence over the long-distance auxin transport (Figure 6, B and I).

We also note that these results showing that ABCBs increase long-distance transport via their co-dependent efflux with the polar PINs also provides strong evidence that this co-dependent efflux is synergistic, rather than antagonistic (as suggested in Blakeslee et al. (2007)). Simulations in a single cell file assuming that ABCB and PIN mediate an antagonistic efflux resulted in a reduction in long-distance auxin transport compared to a case with no ABCB-mediated efflux (Supplemental Figure S14), in contrast to previous long-distance transport measurements in the *abcb* mutant lines (Geisler et al., 2003, 2005; Lewis et al., 2007).

We conclude that considering dynamic auxin distribution provided further insights into the roles of the ABCBs. The modeling showed that ABCBs make a significant contribution to the long-distance auxin transport (as observed experimentally) only provided they enable a polar efflux component, which arises due to synergistic ABCB–PIN-mediated efflux.

## Discussion

Recent review papers have proposed several theoretical scenarios as to whether and how ABCBs and PINs show interactive auxin transport (Spalding, 2013; Geisler et al., 2017). However, an experimental proof of these different scenarios has been hampered by the fact that members of the ABCB and PIN families are functionally redundant and show overlapping transport activities with each other, meaning they function *concertedly* (Geisler et al., 2017). While results from heterologous expression systems have provided support for both independent and interactive ABCB and PIN transport (Bouchard et al., 2006; Blakeslee et al., 2007; Geisler et al., 2017; Deslauriers and Spalding, 2021), results from these may be affected by the presence of endogenous transporters and do not necessarily represent the activity *in planta* (Yesilirmak and Sayers, 2009). In this study, we used a systems approach to investigate potential ABCB–PIN regulatory interactions. Developing a multicellular root-tip model, we predicted how potential ABCB–PIN interactions affect the organ-scale auxin distribution and fluxes. Comparing the predicted and observed DII-VENUS distributions provides a way of assessing which ABCB–PIN interactions function in the root tip.

Predictions in wild-type revealed that ABCB-mediated nonpolar efflux is essential to create low DII-VENUS (high auxin) levels within the outer root layers, as observed experimentally. The nonpolar ABCBs enable auxin to efflux through the periclinal cell membranes into the apoplast where it is subjected to AUX1-mediated influx into specific tissue layers. This suggestion, that ABCBs are able to function as independent auxin catalysts, is supported by a series of heterologous expression studies, especially in nonplant systems, like baker’s yeast and HeLa cells—since these systems typically do not include PINs or PIN-like proteins, these results provide clear evidence for independent ABCB efflux. An independent action is also supported by ABCB localization on the periclinal cell membranes that do not contain PINs. Furthermore, the conclusion that ABCBs efflux auxin independently is also consistent with the evolution of these transporters: while ABCBs are found throughout the plant kingdom, already existing in green algae, PINs functioning as auxin exporters are specific to land plants (Gálvan-Ampudia and Offringa, 2007; Viaene et al., 2014; Zhang et al., 2019). This makes it unlikely that the ABCBs require the presence of PINs to function, as such a scenario would mean they would be unable to function in green algae, making their role unclear.

To further test the interaction scenarios, we simulated a range of auxin-transport mutants; demonstrating the benefits of a modeling approach to test a range of scenarios and identify the key experimental data that would distinguish between the competing hypotheses. The model revealed that the auxin distribution in the *abcb1 abcb19* mutant is affected by the choice of ABCB–PIN interaction scenario.

Predicting the observed *abcb1 abcb19* DII-VENUS distribution required the PINs and ABCBs to mediate a co-dependent efflux, with the PINs mediating either no independent efflux (as in Scenario IV) or a substantially smaller efflux (Scenario III with the co-dependent efflux permeability parameter set to be at least five times larger). We further tested this finding by simulating long-distance transport: in agreement with the mutant results, the predictions only recapitulated previously published experimental results provided ABCBs and PINs contribute to a co-dependent efflux. Such a cooperative mode of ABCB–PIN transport is experimentally supported by protein interaction studies, functional co-expression of PIN1/PIN2 and ABCB1/ABCB4/ABCB19 pairs in Hela and yeast cells, increased electrogenic transport activity where both ABCB4 and PIN2 are present, and analyses of *PIN/ABCB* crosses (Blakeslee et al., 2007; Mravec et al., 2008; Deslauriers and Spalding, 2021). Importantly, in these transport assays, co-expression led to synergistic (more than the sum) transport rates supporting in summary the concept of a *cooperative interaction* (Geisler et al., 2017).

Our model (and results), however, cannot distinguish between the possibilities of the ABCBs acting as regulators of the PINs, or vice versa, the PINs acting as regulators of the ABCBs. It is feasible that ABCB catalyst per se act as a regulator of the PINs, potentially by increasing PIN protein membrane stability (Noh et al., 2003). This concept is supported by examples of mammalian ABC transporters that can, besides functioning as transporters and channels, also act as regulators of secondary active transport systems, including channels (Spalding, 2013; Aryal et al., 2015). A prominent example is the sulfonylurea receptor (SUR/ABCC8;9) that associates with the potassium channel proteins, Kir6.1 or Kir6.2, to form an ATP-sensitive potassium channel (Principalli et al., 2015). Within the channel complex, SUR/ABCC8;9 serves as a regulatory subunit, which fine tunes potassium channel gating.

In further support of our findings, studies in yeast and oocytes have demonstrated that members of the PIN family are able to efflux auxin independently in vitro (Petrášek et al., 2006; Blakeslee et al., 2007; Kim et al., 2010; Henrichs et al., 2012; Zourelidou et al., 2014); however, some members (like PIN1) require posttranscriptional modification provided either by co-expression with an AGC kinase or phosphor-mimikry (Wang et al., 2012; Zourelidou et al., 2014). Interestingly, although baker’s yeast does not contain ABCB, it does contain a subset of ABCGs/PDRs on their plasma membrane and members of their plant orthologs were recently shown to export auxinic compounds, including the auxin precursor IBA but not IAA (Aryal et al., 2019; Ruzicka et al., 2010)—whether the ABCGs/PDRs could be interacting with the PINs to influence PIN-mediated efflux in this system remains to be shown. In this context, it seems important to recall that the original motivation to test interactive ABCB–PIN transport was that in heterologous systems, ABCBs and PINs function as *bona fide* independent auxin transport catalysts but with low specificity and NPA sensitivity that, however, both increased upon co-expression (Blakeslee et al., 2007) providing further experimental support for scenarios in which a co-dependent efflux is present.

The model predictions reproduce the DII-VENUS observations for most single and double ABCB mutants studied; however, differences were present for the *abcb1abcb4* line that we suggest may be caused by the presence of additional ABCBs. Although the model includes the three ABCBs that are established in the root tip, we note that 11 of the 22 full-size ABCBs have recently been suggested to be functional auxin-transporting ABCBs, now being called ATAs (Hao et al., 2020). Adapting the modeling framework to integrate additional ABCBs or alternative ABCB localizations may provide further insights into their roles.

The modeling also demonstrates that polar efflux is essential for long-distance auxin transport. Thus, in our suggested ABCB–PIN interaction scenarios, the lack of the synergistic flux component in the *pin* mutants would contribute to the auxin-related phenotypes observed previously, such as the PIN inflorescences in *pin1* and agravitropic roots in *pin2* (Chen et al., 1998; Gälweiler et al., 1998; Luschnig et al., 1998; Blilou et al., 2005). Previous experimental studies have shown the single *abcb* mutants to have similar phenotypes to wild-type, whereas *abcb1 abcbc19* exhibits altered primary root growth, defective gravitropic bending and twisting of epidermal root files (Geisler et al., 2003; Lin and Wang, 2005; Bouchard et al., 2006; Lewis et al., 2007; Wu et al., 2007, 2010); consistent with our findings that removing single ABCB has only minor effects on the auxin distribution, whereas the root-tip auxin distribution is much perturbed in *abcb1 abcb19*.

Although the model incorporates our current knowledge of the ABCBs and PINs, knowledge of the efflux rates relevant to the different members of the PIN and ABCB family would be beneficial to the field of auxin-transport modeling, although we verified that our conclusions would still hold for wide ranges of these parameter choices (Supplemental Figures S4, S16–S17). Future work applying this systems approach to investigate ABCB–PIN interactions in other plant organs could provide further insights into the role of these interactions in auxin patterning. We note that our conclusions are all drawn from tissue-level analysis of auxin movement, rather than evaluating the transporter function at the level of the membrane; the nature of the suggested functional relationship between ABCBs and PINs at the membrane remains unknown.

In summary, our study provides support for an *interactive* ABCB–PIN action, but how these can be integrated into widely nonoverlapping phenotypes between *PIN* and *ABCB* mutants is still unclear. Future co-expression studies employing combinations of transport-competent and incompetent versions of ABCBs and PINs might be very informative. However, the biochemical gold standard to solve this issue will still require a single and pairwise reconstitution of ABCBs and PINs in a cell-free system.

## Materials and methods

### Plant material and growth conditions

Seeds were surface sterilized with 50% (vol/vol) hypochlorous acid for 5 min and then washed three times with sterile deionized water. Plant seeds were plated on 0.5 strength Murashige and Skoog medium (2.17 g salts per 1 l), at pH 5.8 and solidified with 1% plant agar (Duchefa, Haarlem, The Netherlands). Seeds were stratified at 4°C for 48 h in the dark to synchronize germination, and then incubated vertically in a culture room under 12-h light at 22°C and 12-h dark at 22°C (light: Philips TL-D 58W/840, 120–150 μmol m^−2^ s^−1^). The *Arabidopsis thaliana* ecotype Columbia-0 (Col-0) was used as the wild-type in all experiments. Mutant lines were in the Col-0 background and were obtained from the ABRC and NASC seed repositories for crossing and imaging, lines were genotyped as described in references: we used DII-VENUS (Brunoud et al., 2012), b1-100 (SALK_083649, Lin and Wang, 2005) abcb4: mdr4-1 (SALK_072020, Lewis et al., 2007), and b19-3 (SALK_033455, Lewis et al., 2007).

### RNA extraction and RT-qPCR

Total RNA was extracted from 20 roots using Qiagen RNeasy plant mini kit with on-column DNAse treatment following the manufacturer’s recommended protocol (RNAse-free DNAse Set, Qiagen, Crawley, UK). RNA samples were quantified using a Nanodrop ND100 spectrophotometer (Nanodrop, Wilmington, DE, USA). Poly(dT) complementary DNA (cDNA) was prepared from 2 μg total RNA using the Transcriptor first-strand cDNA synthesis kit (Roche, Basel, Switzerland). Quantitative PCR was performed using SYBR Green Sensimix (Quantace, Mumbai, India) on a Roche LightCycler 480 apparatus. PCR was carried out in 384-well optical reaction plates heated for 1 min to 95°C, followed by 40 cycles of denaturation for 5 s at 95°C, annealing for 8 s at 62°C and extension for 30 s at 72°C. Target quantifications were performed with the specific primer pairs described in Supplemental Table S2. Expression levels were normalized to *ACTIN*. All RT-qPCR experiments were performed in quadruplicates and the values represent mean ± s.e.m.

### Microscopy

Confocal microscopy was performed using a Leica SP8 confocal laser scanning microscope (Leica Microsystems). Cell walls were stained using propidium iodide (10 μg mL^−1^; Sigma-Aldrich, St Louis, MO, USA). Scanning settings used for one experiment were optimized and kept unchanged throughout the experiments.

### Modeling

Root templates were segmented from confocal images using the CellSeT image analysis tool (Pound et al., 2012). We used CellSeT to manually assign a cell type to each cell and then read the geometrical and cell-type data into a tissue database (based on the OpenAlea tissue structure; Pradal et al., 2008). The ABCB distributions were specified based on GFP images (Figure 1, A–C) and PIN, AUX1/LAX, and plasmodesmata distributions were specified as in previous versions of the model (Band et al., 2014; Mellor et al., 2020). These geometrical, topological, and membrane–protein-distribution data were used to form a system of ODEs to describe the auxin transport, synthesis, and degradation within the multicellular root tip. We also simulated a small interaction network model within each cell that describes auxin-mediated DII-VENUS degradation. The parameter values used are based on values in the literature (listed in Supplemental Tables S3 and S4) and we discuss the robustness of our conclusions to these parameter estimates in the “Supplemental Methods.” When comparing the model predictions and experimental data, we focused on the epidermal and cortical cells and subdivided these into the MZ and EZ, respectively, based on a MZ length of 210 μm (obtained as the average MZ length from the multicellular geometries used). Further methodological details about the modeling and equations are provided as “Supplemental Methods.”

### Code availability

Python code that produces each of the simulations is available at https://gitlab.com/nathanmellor/abcbtransport

### Accession numbers

Sequence data from this article can be found in the Arabidopsis Genome Initiative or GenBank/EMBL databases under the following accession numbers:

DII-VENUS (Brunoud et al., 2012)

abcb1: b1-100 (SALK_083649, Lin and Wang, 2005, AT3G28860)

abcb4: mdr4-1 (SALK_072020, Lewis et al., 2007, AT2G47000)

abcb19: b19-3 (SALK_033455, Lewis et al., 2007, AT3G28860).

## Supplemental data 

The following materials are available in the online version of this article.

**Supplemental Methods.** Detailed model description.

**Supplemental Figure S1.** Designated membrane protein distributions.

**Supplemental Figure S2.** Designated cell types in the root-tip templates.

**Supplemental Figure S3**. Predicted wild-type distributions in a geometrically regular root-tip template.

**Supplemental Figure S4.** Quantification of the agreement between model predictions and experimental data for a range of permeability parameter values for wild-type and *abcb1 abcb19*.

**Supplemental Figure S5.** Model predictions and experimental data for an *aux1* mutant.

**Supplemental Figure S6.** Model predictions and experimental data for a *pin2* mutant.

**Supplemental Figure S7.** Model predictions with ABCB4 acting as an influx transporter.

**Supplemental Figure S8.** Replicate DII-VENUS images for double mutant alleles *abcb1-100 abcb4-1*, *abcb4-1 abcb19-1*, and *abcb1-100 abcb19-1*.

**Supplemental Figure S9.** Replicate DII-VENUS images for single mutant alleles *abcb1-100*, *abcb4-1*, and *abcb19-1*.

**Supplemental Figure S10**. Model predictions using Scenario IV for the *abcb* single and double mutants assuming no upregulation of the remaining ABCBs.

**Supplemental Figure S11.** Experimental RT-qPCR measurements of the relative expression of ABCB1, ABCB4, and ABCB19 in the *abcb* single and double mutants.

**Supplemental Figure S12.** Model predictions using Scenario III for the *abcb* single and double mutants assuming a large synergistic efflux (P_SYN_ = 3.0 μm/s).

**Supplemental Figure S13.** Predicted apoplastic auxin concentrations.

**Supplemental Figure S14.** Predicted auxin distributions for the single cell file model showing the effect of the ABCB-independent and co-dependent permeability parameters.

**Supplemental Figure S15.** Predicted long-distance auxin transport assuming a large synergistic efflux (P_SYN_ = 3.0 μm/s).

**Supplemental Figure S16.** Influence of the permeability parameters on the predicted mean auxin concentration in specific tissues in wild-type.

**Supplemental Figure S17**. Influence of the permeability parameters on the predicted mean auxin concentration in specific tissues in the *abcb1 abcb19* mutant.

**Supplemental Table S1.** Cell sizes and numbers used in the geometrically regular template.

**Supplemental Table S2.** Primer pairs used for RT-qPCR.

**Supplemental Table S3**. Parameters for ABCB- and PIN-mediated efflux for each of the five ABCB–PIN interaction scenarios.

**Supplemental Table S4.** Model parameter values with associated references.

**Supplemental Movie S1.** Predictions of rootward long-distance auxin transport for wild-type and Scenario I.

**Supplemental Movie S2**. Predictions of rootward long-distance auxin transport for wild-type and Scenario IV.

**Supplemental Movie S3.** Predictions of rootward long-distance auxin transport for *abcb1 abcb19* and Scenario I.

**Supplemental Movie S4.** Predictions of rootward long-distance auxin transport for *abcb1 abcb19* and Scenario IV.

**Supplemental Movie S5**. Predictions of shootward long-distance auxin transport for wild-type and Scenario I.

**Supplemental Movie S6.** Predictions of shootward long-distance auxin transport for wild-type and Scenario IV.

**Supplemental Movie S7**. Predictions of shootward long-distance auxin transport for *abcb4* and Scenario I.

**Supplemental Movie S8.** Predictions of shootward long-distance auxin transport for *abcb4* and Scenario IV.

## Funding

This work was supported by the Biotechnology and Biological Sciences Research Council (grant number BB/M019837/1) and by the University of Nottingham Faculty of Science Paper Enhancement Fund.

*Conflict of interest statement.* None declared.

## Supplementary Material

koac086_Supplementary_Data

## Notes

L.R.B., U.V., M.J.B., A.B., and D.M.W. designed the research; N.L.M., U.V., A.W., G.J., and D.B. performed research; N.M. and D.B. contributed new computational tools; L.R.B., N.L.M., U.V., A.B., M.J.B., M.G., and D.M.W. analyzed data; L.R.B., N.L.M., U.V., and M.G., wrote the paper with input from all authors.

The author responsible for distribution of materials integral to the findings presented in this article in accordance with the policy described in the Instructions for Authors (https://academic.oup.com/plcell) is Leah R Band (leah.band@nottingham.ac.uk).
