# Supplementary material for: Systems approaches reveal that ABCB and PIN proteins mediate co-dependent auxin efflux
Source: Plant Cell. 2022 Mar 18;34(6):2309–27. doi: 10.1093/plcell/koac086 (PMC9134068; doi:10.1093/plcell/koac086)
Supplement: koac086_Supplementary_Data [file koac086_supplementary_data.zip › Band_00552 PRR.pdf]

Systems approaches reveal that ABCB and PIN proteins mediate co-dependent auxin efflux

Nathan L Mellor, Ute Voß, Alexander Ware, George Janes, Duncan Barrack, Anthony Bishopp, Malcolm J Bennett, Markus Geisler, Darren M Wells, Leah R Band

Corresponding author: Leah R Band leah.band@nottingham.ac.uk

Review timeline:

TPC2020-RA-01028	Submission received:	Dec. 14, 2020
	1st Decision:	Feb. 9, 2021 *manuscript declined*
**TPC2021-RA-00552D**	Submission received:	Nov. 22, 2021
	1st Decision:	Dec. 27, 2021 *accept with minor revisions*
**TPC2021-RA-00552DR1**	1st Revision received:	Jan. 10, 2022
	2nd Decision:	Jan. 10, 2022 *acceptance pending, sent to science editor*
	Final acceptance:	Mar. 10, 2022

REPORT: (The report shows the major requests for revision and author responses. Minor comments for revision and miscellaneous correspondence are not included. The original format may not be reflected in this compilation, but the reviewer comments and author responses are not edited, except to correct minor typographical or spelling errors that could be a source of ambiguity.)

TPC2020-RA-01028 1st Editorial decision – *declined* February 9, 2021

Your submission has been evaluated by members of the editorial board as well as expert reviewers in your field. Overall, we are enthusiastic about this work and the contributions it makes to the field; however, there were some issues that were raised that may require additional experiments and some rethinking. As such, we regret to inform you that we are not able to recommend publication of this manuscript in its current form. We have not made this decision lightly. We have had input from multiple scientists, and we have solicited post-review comments as well. Our present policy is to offer streamlined decisions and to not advise on the direction of the work by requesting extensive modifications or substantial additional experiments.

During the post-review consultation session, we also agreed that if you could address the major points raised by the reviewers by new experiments, we would welcome a resubmission. This may be treated as a new submission, but we would attempt to use at least some of the same reviewers. Nevertheless, reviewers may be asked to assess as a new manuscript (i.e. are the claims fully supported by the data and do the results presented move the field forward?), and not only whether previous reviewer comments have been addressed.

Both reviewers were highly enthusiastic about the potential contributions this work makes to our understanding of the relationship between the PIN and ABCB proteins; however, both raised distinct points that need to be fully addressed before they can adequately assess the new models.

Reviewer #1 in particular stated an inability to properly assess the work without clarification of the input used for scenario IV (the first point listed for Reviewer #1).

-- Reviewer comments:

Reviewer #1 (Comments for the Author):

This paper presents a revised and expanded model of auxin transport in the root that includes various combinations of PIN, ABCB, and PIN-ABCB synergistic interactions working in roots with appropriately shaped cells. It is exciting to see that models of this nature continue to evolve, and the comparison to DII-Venus in living roots, where provided, is a strength of the paper. The primary claim of the paper is that the modeling scenario that best predicts living plants (scenario IV) is one in which PINs only work in combination with ABCBs; that is, that PINs do not work alone. This is a surprising result given prior work showing that PINs function independently in heterologous systems, though it is

possible that other protein(s) operate as co-transporters in those systems. At present, two factors prevent me from properly assessing the extent to which the claim in this work is supported.

1. I don't understand the scenarios. In Figure 2, it appears that scenario IV is based on a combination of ABCBs working alone and in synergistic combination with PINs. In Table S2, it appears that scenario IV models ABCBs working alone, and that there is no synergistic interaction with PINs. Clarification is required.

2. All of the roots shown within a figure should have the same color scale. In Fig, 2, I can not currently assess to extent to which the lack of auxin accumulation in the epidermis in scenarios 2 and 5 is due to the change in scale. Retaining one scale is especially important in Fig. 3 where all roots are compared against a single standard (Dll-Venus). If it is not possible to show the desired level of detail in some roots when all are set to one scale, add columns to show the roots at a second scale as well-similar to the inclusion of an inset on a photograph.

Other comments and editorial suggestions:

Please include a sentence in the main text indicating the relative strength of synergistic interactions, as compared to individual activities (that is, please summarize Table S2 in the main text).

The color scale bar(s) next to the model roots should be larger.

In addition, the color map here is not perceptually uniform. It would be appreciated if you used colormap viridis, or another perceptually uniform colormap.

(https://journals.plos.org/plosone/article?id=10.1371/journal.pone.0199239)

The paper might work better if Figure 2 were limited to a diagram of the very simple model that forms the basis for part K, and those results, with the rest of that figure moved to the supplement. Then you could go on to discuss validation of the detailed model in Figure 3. If you prefer to go into a discussion about which geometrical model is best, then please include an illustration showing what distribution is present in a live plant so that readers can fully consider the extent to which each model may be validated.

Data in Fig. 6 cannot be properly evaluated because the paper lacks a clear indication of what the result in a living organism looks like. Adding auxin to your existing Dll lines and showing the result an hour later, or getting permission to reprint results from other experiments, would greatly strengthen this section.

Within a figure, were all the Dll images taken with the same settings? Please clarify.

Early in the introduction, please state that you are talking about Arabidopsis thaliana.

Line 186 abcb1abcb1-> abcb1abcb19?

Line 416 "Model reveals that ABCB4 does not function as an auxin influx carrier in the root tip"

should be changed to "Model predicts that ABCB4 does not function as an auxin influx carrier in the root tip," because the model can only make suggestions about what may be true.

In the legend for Fig 3, when you say "Predicted wild type auxin distribution for the five ABCB-PIN interaction scenarios," do you mean: Wild type auxin distribution produced by running each of the five ABCB-PIN interaction scenarios? If so, rephrasing along these lines would helpful.

Line 452-454, please rephrase

Line 596 (currently blank). The story would be easier to follow if you briefly stated, at this point, what the experimental result is when auxin is added to the shoot. An outcome is mentioned at line 603-604, but it is buried in the description of the model results, which makes it difficult to compare. Also, it is not clear from the text if that result is from abcb1and abcb19 separately, or from the double mutant.

Again around line 621, it would be helpful to briefly remind the reader of what the experimental data show when auxin is deposited at the root tip in wildtype and the different abcb mutants. You have this in the introduction, but many readers will have forgotten the details by the time they reach this point.

Use of the verb 'predicted' is often confusing because I think you sometimes use this word to describe what the output of a simulation was, and sometimes what you expect that the output might be. In the opening sentence of line 621, I think you are talking about what you expected that your simulations would show. You could clarify this by saying: We expected that scenarios...would show...

Gälweiler et al. in the references needs to be reformatted.

Reviewer #2 (Comments for the Author):

The manuscript from Mellor et al. provides a thoughtful comparison of the modelling of five carefully chosen scenarios for auxin transport in the root tip with selected experimental results. The criteria establish for evaluation and the outputs that are tested are well thought out and are compelling. The manuscript is very well written, it is was truly a delight to read and review. There are a few fungus gnats in the Petri dish to consider:

1. The conclusions drawn are valid in the context of a tissue-level analysis of auxin movement, but may not be accurate at the level of the cell surface. The model deals with auxin inside the cell, auxin that is outside the cell, and even auxin moving to an adjacent cell. However, transporter function at the level of the membrane is not really evaluated. As such, the conclusion come a bit too close to stating that PINS are likely not transporting auxin and ABCBs likely are, when it is quite possible that an interdependent functionality not visible at the granularity of the models and experiments may be in play. The authors might want to make this clear.

2. The discrepancies between the predicted and measured abcb1 and abcb1abcb4 auxin distributions could be explained by compensatory upregulation of ABCB19 in abcb1 (Jenness et al., 2019) which may be greater in the abcb1abcb4 double. This could be addressed with assessments of abcb1 and abcb4 conducted in the abcb19 background. Also important to indicate the alleles in all cases. Hopefully, all are in the Col-0 background.

3. The manuscript states that ABCB4 is not expressed in the columella. Although expressed to varying extents, ABCB4 signals are detected vi GUS staining, immunolocalization, and analysis of both ABCB4-GFP and ABCB4-YFP. Further, although the authors correctly cite Kubes et al., 2012 in noting that ABCB4 functions exclusively as an exporter except with very low nM auxin levels and the export function appears to be the primary contribution of ABCB4 to shootward auxin transport streams, the same figure in the same paper clearly shows a contribution to transport of auxin from the columella. This should be addressed.

Overall, this is an excellent paper that makes a very important contribution to the field and to general knowledge. The authors are to be congratulated on its innovation and thoroughness. Further, it is one of the most readable manuscripts I have encountered in quite some time. With the issues mentioned addressed, it could certainly be a notable Plant Cell paper.

**TPC2021-RA-00552D Submission received**	**November 22, 2021**

Reviewer comments on previously declined manuscript and **author responses:**

> **We thank the reviewers for their detailed consideration of this manuscript and constructive comments. We have carefully considered each of these suggestions and addressed these inturn, as described in the document below. These suggestions have led to us substantially improving the clarity and structure of the manuscript text and figures, as well as integrating new qRT–PCR data to quantify the *ABCB1, 4* and *19* expression in the *abcb* single and doublemutants studied. Furthermore, while preparing this resubmission, we also performed exhaustive searches of parameter space which revealed an additional scenario in which the model predictions agree with the data. This scenario had a larger co-dependent efflux where both ABCB and PIN are present, but also a low level of PIN independent efflux (less than 15% of the total efflux). This addition to the manuscript provides further support for our conclusionthat a co-dependent ABCB-PIN efflux is essential to auxin dynamics and that PIN-mediated efflux is predominantly through this co-dependent efflux component.**

Reviewer #1 (Comments for the Author):

This paper presents a revised and expanded model of auxin transport in the root that includesvarious combinations of PIN, ABCB, and PIN-ABCB synergistic interactions working in roots with appropriately shaped cells. It is exciting to see that models of this nature continue to evolve, and the comparison to DII-Venus in living roots, where provided, is a strength of the paper. The primary claim of the paper is that the modeling scenario that best predicts living plants (scenario IV) is one in which PINs only work in combination with ABCBs; that is, that PINs do not work alone. This is a surprising result given prior work showing that PINs functionindependently in heterologous systems, though it is possible that other protein(s) operate as co-transporters in those systems. At present, two factors prevent me from properly assessingthe extent to which the claim in this work is supported.

I don't understand the scenarios. In Figure 2, it appears that scenario IV is based on a combination of ABCBs working alone and in synergistic combination with PINs. In Table S2,it appears that scenario IV models ABCBs working alone, and that there is no synergistic interaction with PINs. Clarification is required.

We apologise that there was a mistake in the Table S2 (now labelled Table S3 in the new version), which we have now corrected.

All of the roots shown within a figure should have the same color scale. In Fig, 2, I cannot currently assess to extent to which the lack of auxin accumulation in the epidermis in scenarios2 and 5 is due to the change in scale. Retaining one scale is especially important in Fig. 3 where all roots are compared against a single standard (DII-Venus). If it is not possible to showthe desired level of detail in some roots when all are set to one scale, add columns to show the roots at a second scale as well-similar to the inclusion of an inset on a photograph.

To select amongst the scenarios, we compare model predictions with DII-VENUS observations, focussing on the predicted and observed distribution patterns. Using the distribution patterns provides the most information, because looking at absolute levels tells us very little about which scenario is correct: the DII-VENUS experimental data is showing us which of the patterns are correct, and not which absolute level is correct. We therefore use a scale to best show and compare the distribution patterns, even if this results in using different scales for different scenarios. The predicted auxin concentrations are relative to the stele concentration at the boundary of the modelled root tissue, to ensure we focus on the modelled dynamics in the root tip region, rather than any influence of the amount reaching the root tip from the shoot.

Other comments and editorial suggestions:

Please include a sentence in the main text indicating the relative strength of synergistic interactions, as compared to individual activities (that is, please summarize Table S2 in themain text).

We have added these details, see Line 253.

The color scale bar(s) next to the model roots should be larger.

We have doubled the size of the colour scale bars.

In addition, the color map here is not perceptually uniform. It would be appreciated if youused colormap viridis, or another perceptually uniform colormap. (https://journals.plos.org/plosone/article?id=10.1371/journal.pone.0199239)

Thank you for this suggestion. We now show all results with the viridis colour map, assuggested, and agree that the results are clearer with this choice.

The paper might work better if Figure 2 were limited to a diagram of the very simple model that forms the basis for part K, and those results, with the rest of that figure moved to the supplement. Then you could go on to discuss validation of the detailed model in Figure 3. If you prefer to go into a discussion about which geometrical model is best, then please includean illustration showing what distribution is present in a live plant so that readers can fully consider the extent to which each model may be validated.

We agree that the structure of these first sections could be improved. We have moved Fig 2A-J in the original submission into the Supplementary material (Figure S3), and now focus the main text of the

simulations using the real multicellular root-tip templates. Furthermore, we have moved Fig 2K (from the original manuscript) together with a new diagram of the simple single-cell-file model to form a new Figure 5; and have moved the associated description to be the penultimate subsection of the Results in order to use these ideal-geometry simulation results to clarify and motivate the findings about long-distance transport.

Data in Fig. 6 cannot be properly evaluated because the paper lacks a clear indication of whatthe result in a living organism looks like. Adding auxin to your existing DII lines and showing the result an hour later, or getting permission to reprint results from other experiments, wouldgreatly strengthen this section.

We have integrated previously published data on long-distance transport into this figure and now focus the model results on these cases, which makes the conclusions clearer. More specifically, Figure panels 6C and 6J now show previously published data from a Plant Cell paper (Lewis et al Plant Cell, 19: 1838–1850 2007) which presented measurements of long- distance auxin transport within the root (although other long-distance transport data have beenpublished previously, these focus on other plant organs). The corresponding author of this paper, Prof Edgar Spalding, has agreed for us to integrate his data in this way.

Within a figure, were all the DII images taken with the same settings? Please clarify.

Yes, the scanning settings were kept unchanged throughout the experiments; this has now been added to the experimental methods.

Early in the introduction, please state that you are talking about Arabidopsis thaliana.

We have added this detail to the first paragraph of the introduction (see Line 75).

Line 186 abcb1abcb1-> abcb1abcb19?

This has been changed.

Line 416 "Model reveals that ABCB4 does not function as an auxin influx carrier in the root tip" should be changed to "Model predicts that ABCB4 does not function as an auxin influx carrierin the root tip," because the model can only make suggestions about what may be true.

This has been changed (see Line 372 in the revised version).

In the legend for Fig 3, when you say "Predicted wild type auxin distribution for the five ABCB-PIN interaction scenarios," do you mean: Wild type auxin distribution produced by running each of the five ABCB-PIN interaction scenarios? If so, rephrasing along these lines would helpful.

We have rephrased this, as suggested (note that these results are now Figure 2 in the revisedmanuscript).

Line 452-454, please rephrase

We agree that these lines were a little clumsy and have rephrased them, as suggested (see Lines 405-407).

Line 596 (currently blank). The story would be easier to follow if you briefly stated, at this point,what the experimental result is when auxin is added to the shoot. An outcome is mentioned atline 603-604, but it is buried in the description of the model results, which makes it difficult to compare. Also, it is not clear from the text if that result is from abcb1and abcb19 separately, or from the double mutant.

We fully agree and have added these details, as suggested, see Line 587 in the revised manuscript.

Again around line 621, it would be helpful to briefly remind the reader of what the experimentaldata show when auxin is deposited at the root tip in wildtype and the different abcb mutants. You have this in the introduction, but many readers will have forgotten the details by the timethey reach this point.

We have added these details, as suggested, see Line 607 in the revised manuscript.

Use of the verb 'predicted' is often confusing because I think you sometimes use this word todescribe what the output of a simulation was, and sometimes what you expect that the output might be. In the opening sentence of line 621, I think you are talking about what you expectedthat your simulations would show. You could clarify this by saying: We expected thatscenarios...would show...

We have carefully checked the manuscript to ensure that we use the terms 'predicted' and 'expected' in appropriate places. Furthermore, throughout the manuscript we now use the term'the model predicted' rather than 'we predicted' to avoid confusion.

Gälweiler et al. in the references needs to be reformatted.

We have corrected this.

Reviewer #2 (Comments for the Author):

The manuscript from Mellor et al. provides a thoughtful comparison of the modelling of five carefully chosen scenarios for auxin transport in the root tip with selected experimental results.The criteria establish for evaluation and the outputs that are tested are well thought out and are compelling. The manuscript is very well written, it is was truly a delight to read and review.There are a few fungus gnats in the Petri dish to consider:

The conclusions drawn are valid in the context of a tissue-level analysis of auxin movement, but may not be accurate at the level of the cell surface. The model deals with auxin inside thecell, auxin that is outside the cell, and even auxin moving to an adjacent cell. However, transporter function at the level of the membrane is not really evaluated. As such, the conclusion come a bit too close to stating that PINS are likely not transporting auxin and ABCBs likely are, when it is quite possible that an interdependent functionality not visible at the granularity of the models and experiments may be in play. The authors might want to makethis clear.

We agree that this is an important point, and we have added this to the penultimate paragraphof the discussion section (see Lines 747-750).

The discrepancies between the predicted and measured abcb1 and abcb1abcb4 auxin distributions could be explained by compensatory upregulation of ABCB19 in abcb1 (Jenness et al., 2019) which may be greater in the abcb1abcb4 double. This could be addressed with assessments of abcb1 and abcb4 conducted in the abcb19 background. Also important to indicate the alleles in all cases. Hopefully, all are in the Col-0 background.

Motivated by this suggestion, we performed qRT–PCR to quantify the expression of the threeABCBs in each single and double mutant. As hypothesised by this reviewer, we did indeed observe compensatory upregulation of ABCB19 in *abcb1* and *abcb1abcb4* (see new Supplementary Figure S11). Integrating these fold changes into the model, we found only minor changes in the predicted auxin and DII-VENUS distributions. These new results are described in the main text, Lines 488-495.

The Columbia (Col-0) ecotype was used for all experiments; we have now provided details ofthe alleles and ecotype in the Methods section.

The manuscript states that ABCB4 is not expressed in the columella. Although expressed to varying extents, ABCB4 signals are detected vi GUS staining, immunolocalization, and analysis of both ABCB4-GFP and ABCB4-YFP. Further, although the authors correctly cite Kubes et al., 2012 in noting that ABCB4 functions exclusively as an exporter except with verylow nM auxin levels and the export function appears to be the primary contribution of ABCB4to shootward auxin transport streams, the same figure in the same paper clearly shows a contribution to transport of auxin from the columella. This should be addressed.

Thank you for highlighting this, as suggested we have now incorporated ABCB4 into the columella in the model, and have included the citation to Kubes et al. 2012 as suggested (seeFigure 1B,D and Lines 142, 196 and 199).

Overall, this is an excellent paper that makes a very important contribution to the field and to general knowledge. The authors are to be congratulated on its innovation and thoroughness.Further, it is one of the most readable manuscripts I have encountered in quite some time. With the issues mentioned addressed, it could certainly be a notable Plant Cell paper.

Many thanks for your suggestions and positive comments, we hope that you find the revised manuscript improved.

TPC2021-RA-00552D 1st Editorial decision – *accept with minor revisions* December 27, 2021

On the basis of the advice received, the board of reviewing editors would like to accept your manuscript for publication in The Plant Cell. This acceptance is contingent on revision based on the comments of our reviewers. In particular, please address the minor concerns of Reviewer #1, which point out a few sentences that require clarification and asks that scales in Figures 3 and 4 were uniform to make comparisons easier.

-- Reviewer comments:

Reviewer #1 (Comments for the Author):

Thank you for the highly responsive set of revisions, which have made the paper much stronger.

In Fig 6, the color bars associated with the different simulations all have the same maximum value. This is excellent and makes comparison easy. I wish that the scale bars in Figs 3 and 4 were also set up this way, as it would greatly facilitate cross-genotype comparisons.

A few smaller scale comments:

I did not understand the sentences around line 467-469. I went to Figure 4 and was able to figure out from the legend and the supplement what it shows, but I didn't understand what you were pointing out in the text. The information I was expecting earlier arrived around line 486.

Line 494-496-I think the extent of agreement is over stated. The abcb 19 and abcb1 abccb19 double do look good, but abcb 4 is quite far off.

Are the images shown in Fig 3S and Fig 4U-- and Fig 3T and Fig 4V--the same? If they are not identical, a statement as to how the images differ should be added. If they are identical, I offer the friendly reminder that the figure legends are required to make it clear when identical data is shown in more than one location.

Line 588-9 I am not sure that I follow. What is it that has equal permeability: PIN and ABCB alone? Are you saying that you ran scenario I, or saying something else?

Line 598: "the model predictions with scenario I are clearly inconsistent with these data (Figure 6C). Therefore, including a co-dependent ABCB-PIN mediated efflux is essential for predictions to agree with the observations"

The second half of this does not automatically follow from the first-theoretically, many aspects of the model could be result in scenario I not working, without co-dependent efflux being required. Please rephrase. (I am not objecting to your results-your simulations show good agreement with scenario iv; I am just objecting to the wording.)

Reviewer #2 (Comments for the Author):

The revised manuscript presented by the authors satisfies the concerns that I had in the first review. I also feel that the authors have satisfied the requests of the other reviewers, with one minor exception. In addressing the comment that the model doesn't support ABCB4-mediated auxin uptake in the root apex, the authors should refine their statement to by adding " and at the auxin concentrations present at the root tip". This is more consistent with the conditions used in the modelling as well as previously -published physiological data suggesting that ABCB4 can only act in auxin uptake under very low auxin concentrations.

Reviewer comments on previous submission and **author responses**:

Reviewer #1:
Thank you for the highly responsive set of revisions, which have made the paper much stronger.
In Fig 6, the color bars associated with the different simulations all have the same maximum value. This is excellent and makes comparison easy. I wish that the scale bars in Figs 3 and 4 were also set up this way, as it would greatly facilitate cross-genotype comparisons.

We had previously considered setting up the colour bar maximum for the mutant predictions (Figs 3 and 4) in the way suggested. However, this choice would imply that the concentrations presented for one mutant are comparable to those of another mutant. In fact, these concentrations are all relative to the auxin concentration in the cells at the boundary of the modelled tissue, and so depend on the influx of auxin from the shoot. In the mutants, the long-distance auxin transport from shoot-to-root is affected; given we have insufficient data to quantify and integrate this into the steady-state root tip predictions at this stage, it is therefore most accurate to focus the comparisons between steady-state root-tip predictions and data on the patterns, rather than the absolute level. Hence, we would prefer to keep the colour bar maximums relevant to showing the patterns.

A few smaller scale comments:
I did not understand the sentences around line 467-469. I went to Figure 4 and was able to figure out from the legend and the supplement what it shows, but I didn't understand what you were pointing out in the text. The information I was expecting earlier arrived around line 486.

We agree that the information in lines 467-469 was misplaced, and that the data in these figure panels was explained more clearly later. We have therefore removed lines 467-469.

Line 494-496-I think the extent of agreement is over stated. The abcb 19 and abcb1 abccb19 double do look good, but abcb 4 is quite far off.

We agree, and have changed the text as follows:

Comparing the predicted DII-VENUS patterns with those observed, we found reasonable agreement between predictions and data in *abcb19, abcb4abcb19* and *abcb1abcb19*, although some differences for *abcb1*, *abcb4* and *abcb1abcb4* (Figure S10).

Are the images shown in Fig 3S and Fig 4U-- and Fig 3T and Fig 4V--the same? If they are not identical, a statement as to how the images differ should be added. If they are identical, I offer the friendly reminder that the figure legends are required to make it clear when identical data is shown in more than one location.

Thank you for highlighting this. We have added this information to the figure legend, as follows:

"We note that to aid readability, the data presented in Fig 3S,T is repeated here in panels 4U,V."

Line 588-9 I am not sure that I follow. What is it that has equal permeability: PIN and ABCB alone? Are you saying that you ran scenario I, or saying something else?

We agree that this sentence was unclear, and we have rewritten it to clarify the model assumptions used in these simulations, as follows:

"Motivated by the findings from the steady-state distributions, we performed simulations both with scenario IV, with the ABCB-independent and ABCB-PIN co-dependent efflux having equal permeabilities (Figure 6), and with scenario III, with a large co-dependent permeability and small permeabilities for ABCB and PIN independent efflux (Figure S15)."

Line 598: "the model predictions with scenario I are clearly inconsistent with these data (Figure 6C). Therefore, including a co-dependent ABCB-PIN mediated efflux is essential for predictions to agree with the observations"

The second half of this does not automatically follow from the first-theoretically, many aspects of the model could be result in scenario I not working, without co-dependent efflux being required. Please rephrase. (I am not objecting to your results-your simulations show good agreement with scenario iv; I am just objecting to the wording.)

We agree that this was unclear, and have now rewritten the sentence as follows:

"…the model predictions with scenario I are clearly inconsistent with these data, whereas including a co-dependent ABCB-PIN mediated efflux can result in model predictions that agree with the observations (Figure 6C)."

Reviewer #2 (Comments for the Author):
The revised manuscript presented by the authors satisfies the concerns that I had in the first review. I also feel that the authors have satisfied the requests of the other reviewers, with one minor exception. I addressing the comment that the model doesn't support ABCB4-mediated auxin uptake in the root apex, the authors should refine their statement to by adding " and at the auxin concentrations present at the root tip". This is more consistent with the conditions used in the modelling as well as previously published physiological data suggesting that ABCB4 can only act in auxin uptake under very low auxin concentrations.

We fully agree with this suggestion and have added this detail to lines 367, which now states:

" … supposing that ABCB4 acts as an influx transporter at the auxin concentrations present at the root tip."

**TPC2021-RA-00552DR1 2nd Editorial decision – *acceptance pending***	**January 10, 2022**

We are pleased to inform you that your paper entitled "Systems approaches reveal that ABCB and PIN proteins mediate co-dependent auxin efflux" has been accepted for publication in The Plant Cell, pending a final minor editorial review by journal staff. At this stage, your manuscript will be evaluated by a Science Editor with respect to its presentation of scientific content, compliance with journal policies, and presentation for a broad readership.

**Final acceptance from Science Editor**	**March 10, 2022**
