# Supplementary material for: Systems approaches reveal that ABCB and PIN proteins mediate co-dependent auxin efflux
Source: Plant Cell. 2022 Mar 18;34(6):2309–27. doi: 10.1093/plcell/koac086 (PMC9134068; doi:10.1093/plcell/koac086)
Supplement: koac086_Supplementary_Data [file koac086_supplementary_data.zip › SI_final_files.pdf]

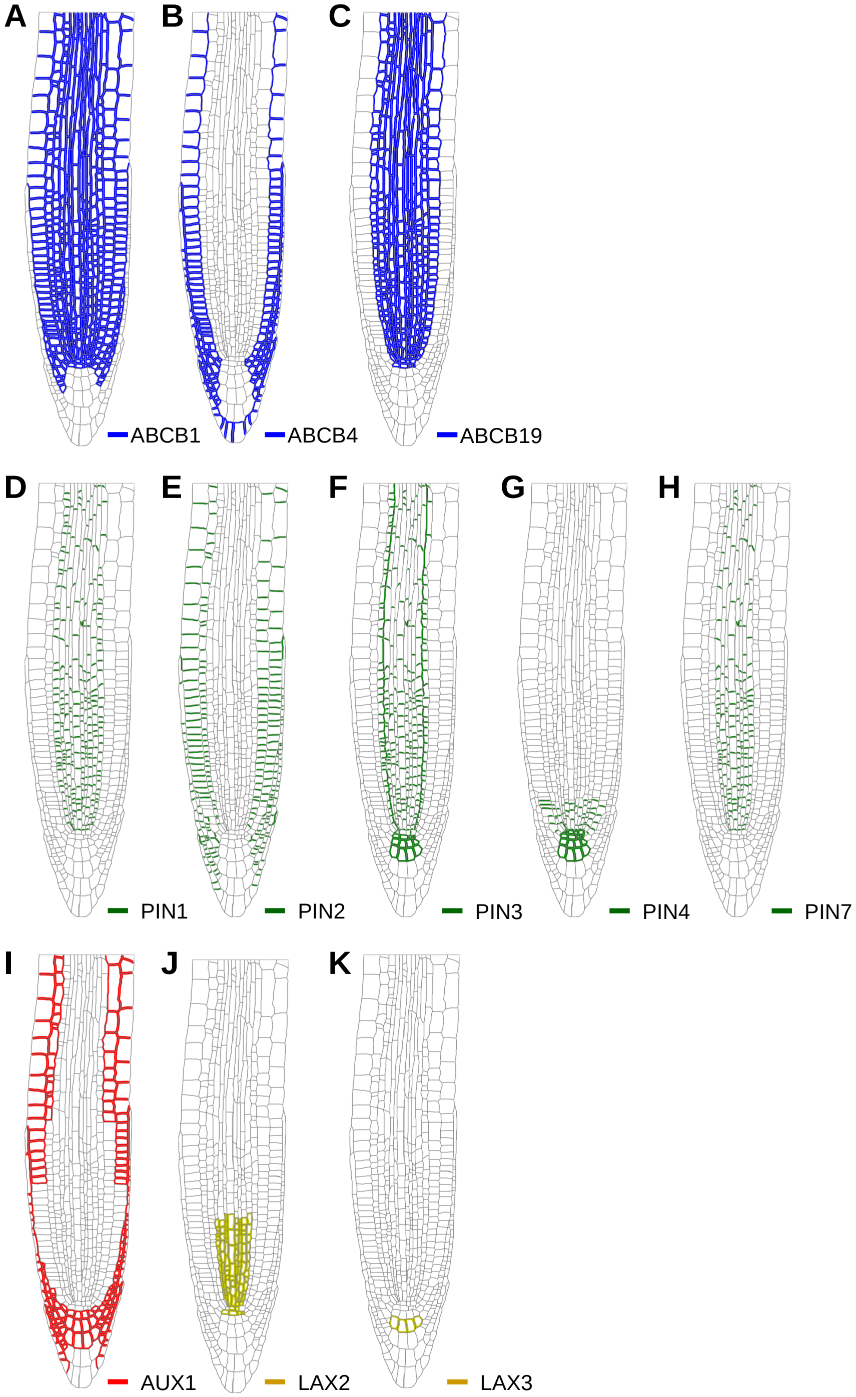

Supplemental Figure 1: Designated membrane protein distributions.
(Supports Figure 1.)
(A) ABCB1, (B) ABCB4, (C) ABCB19, (D) PIN1, (E) PIN2, (F) PIN3, (G) PIN4, (H) PIN7, (I) AUX1, (J) LAX2, (K) LAX3.

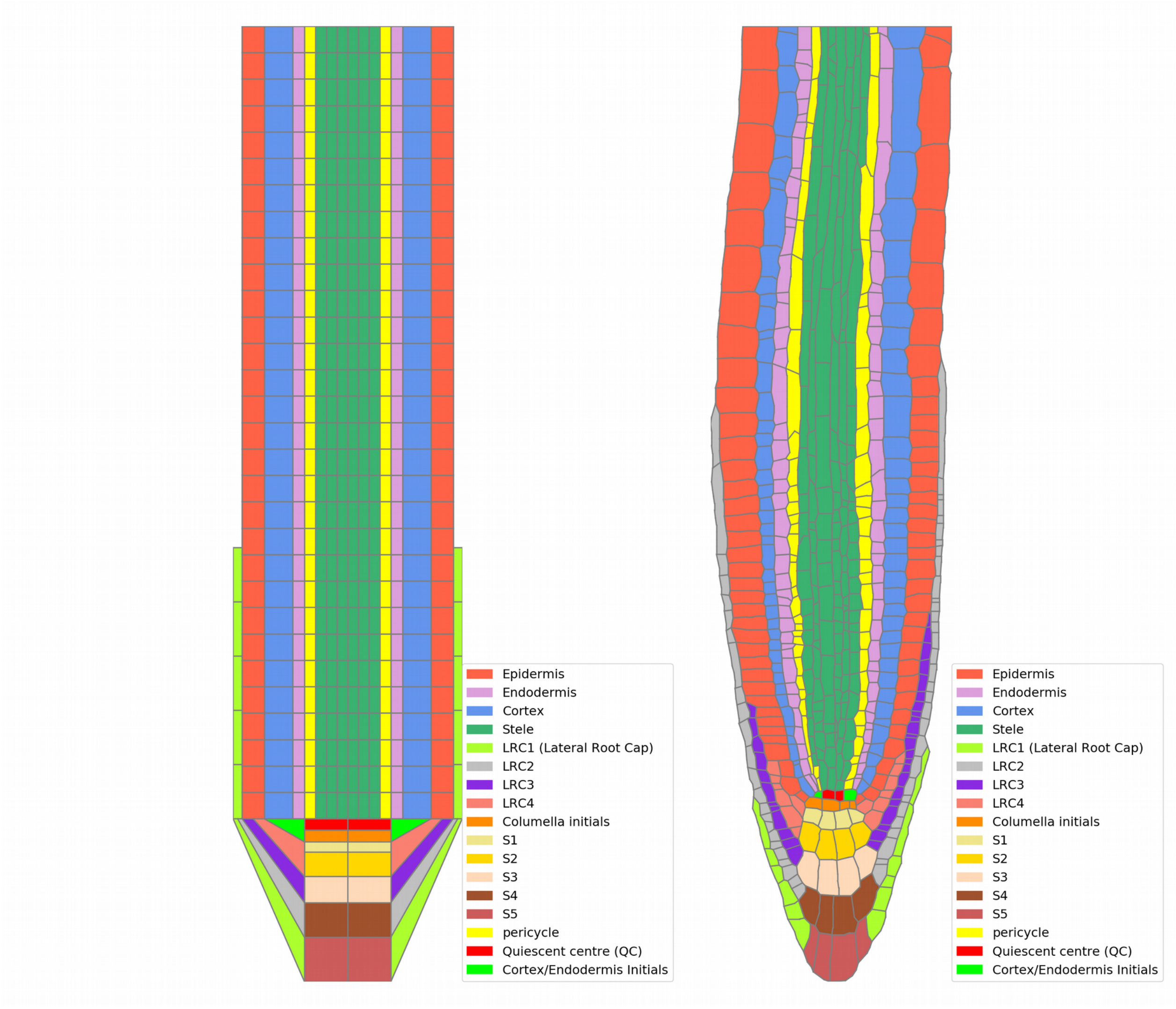

Supplemental Figure 2: Designated cell types in the root-tip templates.
(Supports Figure 1.)
(A) Geometrically regular multicellular template.
(B) Real multicellular template segmented from the confocal image in Figure 2U.

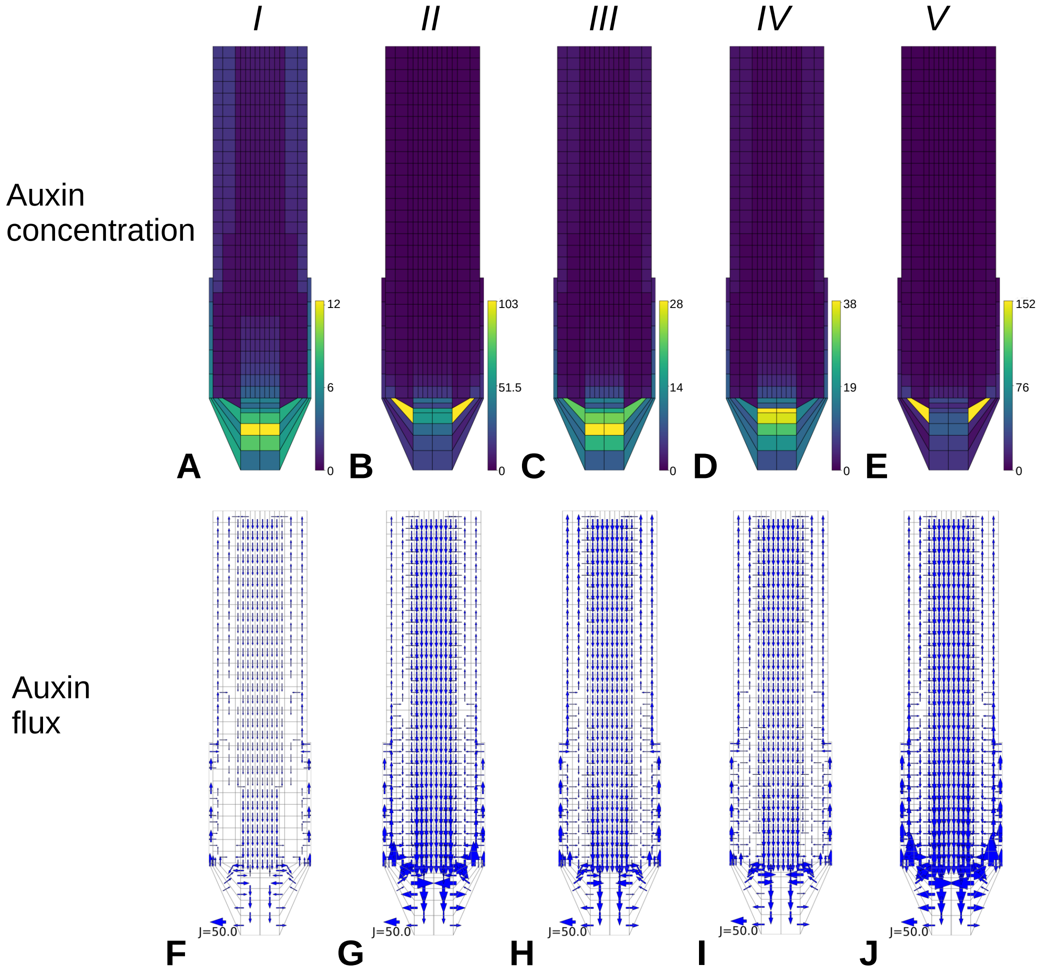

Supplemental Figure 3: Predicted wild-type distributions in a geometrically regular root-tip template. (Supports Figure 1.)
(A-E) Predicted auxin concentrations for each of the five ABCB-PIN interaction scenarios (detailed in Figure 1F).
(F-J) Predicted auxin fluxes for each of the five ABCB-PIN interaction scenarios (detailed in Figure 1F).

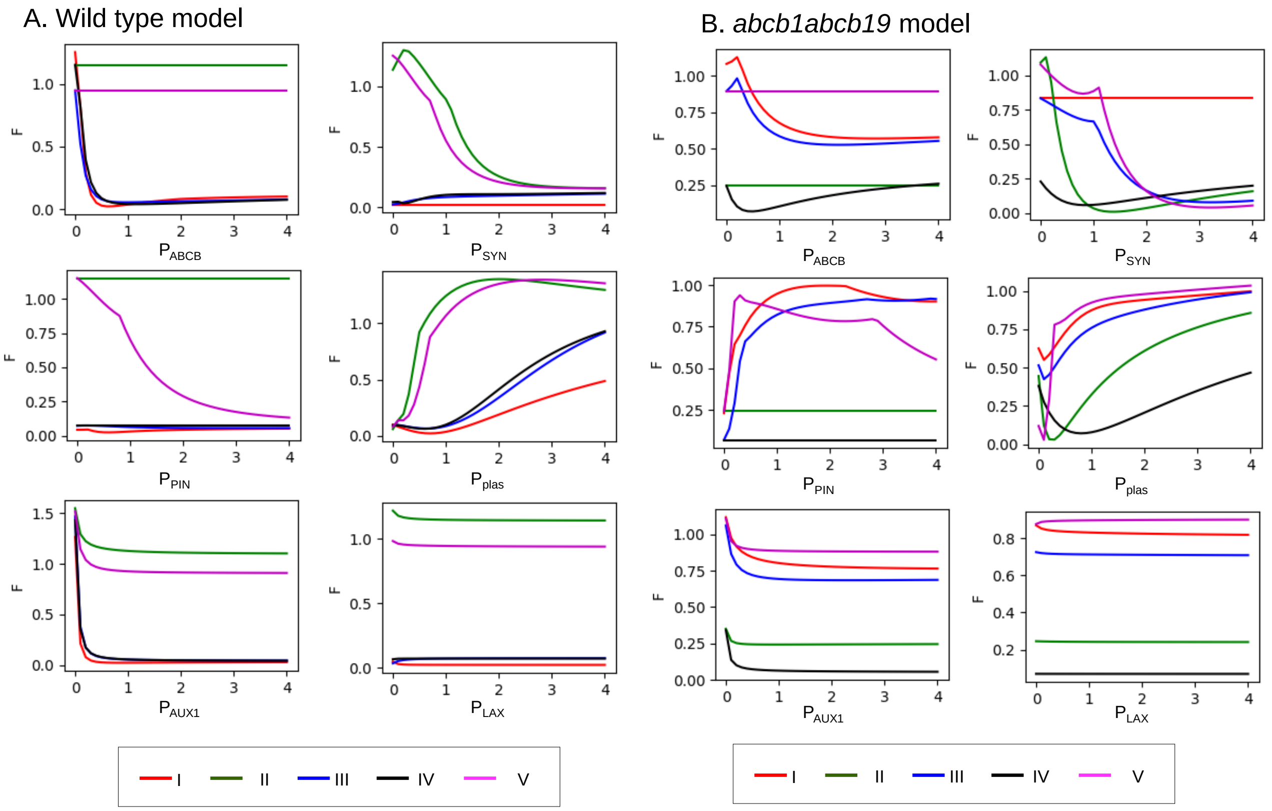

Supplemental Figure 4: Quantification of the agreement between model predictions and experimental data for a range of permeability parameter values for wild type and *abcb1 abcb19*.
(Supports Figures 2 and 3.)
(A) Values of fit function, F, for wild type in each scenario I-V for a range of values for the permeability parameters P_{ABCB}, P_{SYN}, P_{PIN}, P_{plas}, P_{AUX1} and P_{LAX}.
(A) Values of fit function, F, for *abcb1 abcb19* in each scenario I-V for a range of values for the permeability parameters P_{ABCB}, P_{SYN}, P_{PIN}, P_{plas}, P_{AUX1} and P_{LAX}.
We note that lower values of F represent a better fit between mean values in the epidermis and cortex. These results suggest that predictions with scenarios I,III and IV are in good agreement with the data in wild type for wide ranges of each parameter. Considering the *abcb1abcb19* simulations, we see that predictions in scenario IV are in best agreement in most cases, although scenario III predictions are in best agreement with large values of P_{SYN}.

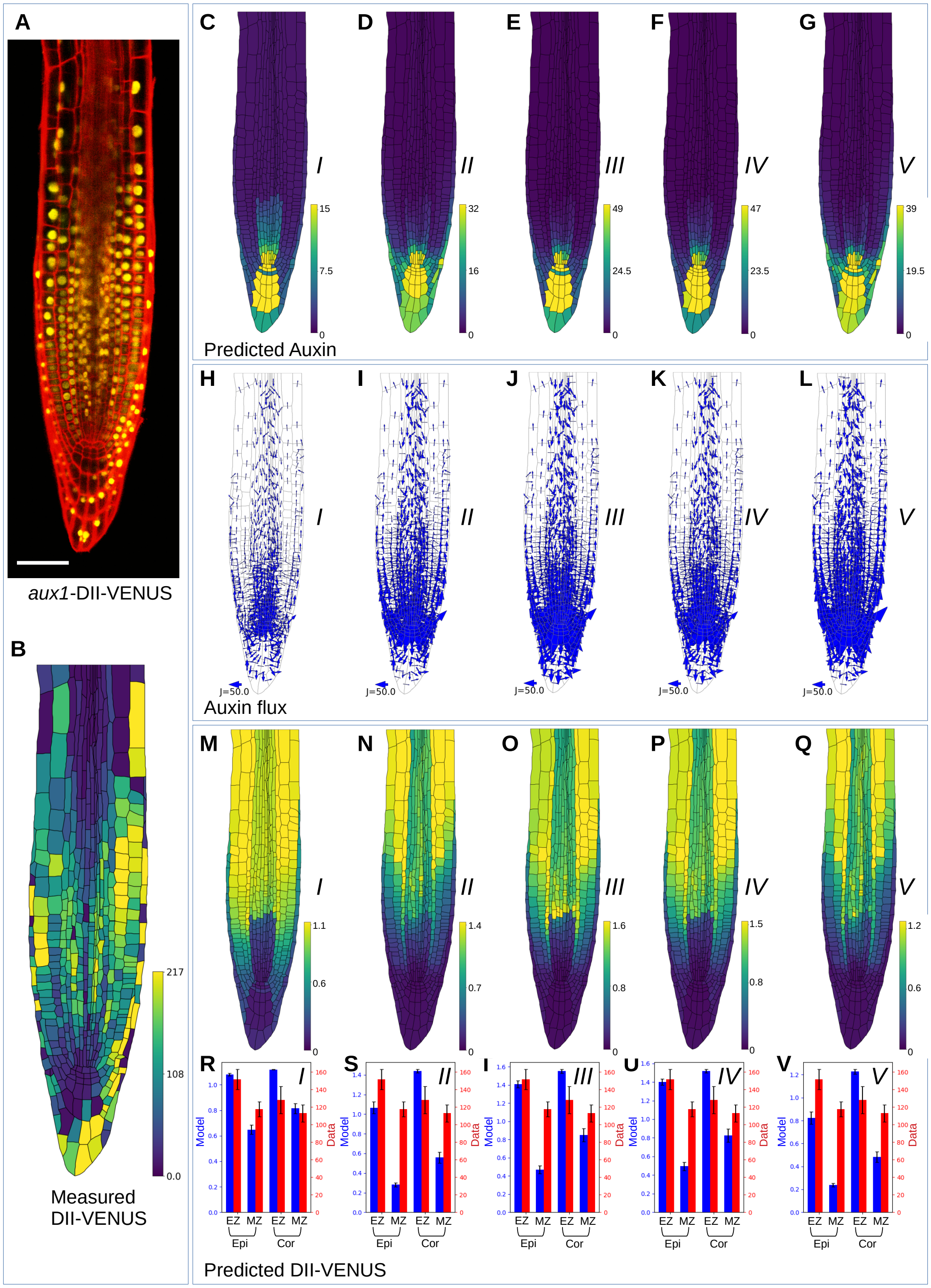

Supplemental Figure 5: Model predictions and experimental data for an *aux1* mutant. (Supports Figure 2.)
(A) Confocal image of an *aux1-DII-VENUS* root tip, reporting DII-VENUS (yellow) and propidium iodide staining (red).
(B) Measured DII-VENUS levels extracted from the confocal image in (A).
(C-G) Predicted auxin concentrations for each of the five ABCB-PIN interaction scenarios.
(H-L) Predicted auxin fluxes for each of the five ABCB-PIN interaction scenarios.
(M-Q) Predicted DII-VENUS concentrations for each of the five ABCB-PIN interaction scenarios.
(R-V) Mean DII-VENUS in the meristematic and elongation-zone regions of the epidermis and cortex, comparing the model predictions in each of the five ABCB-PIN interaction scenarios with the experimental data. Error bars show ±1 s.e.m.

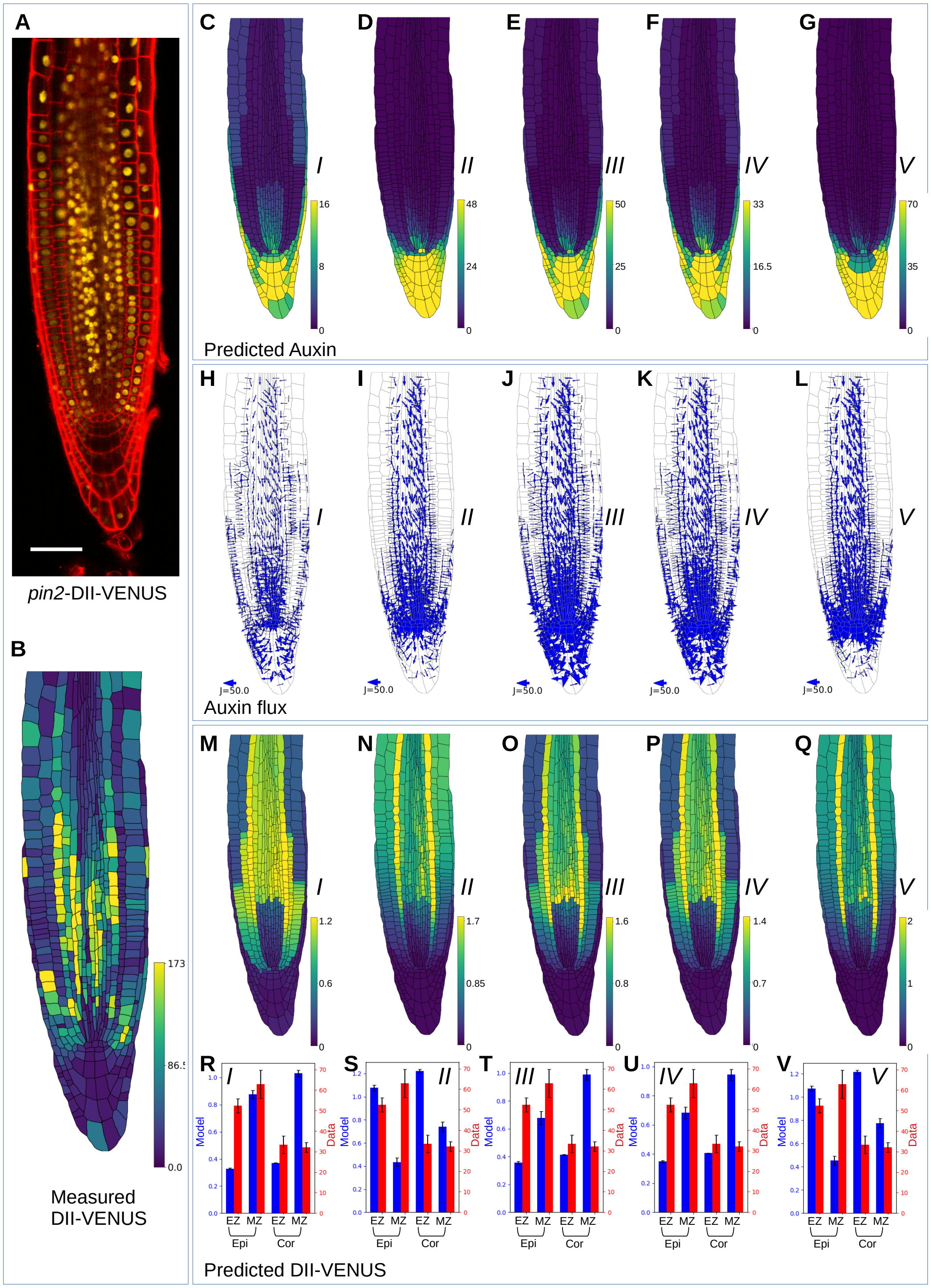

Supplemental Figure 6: Model predictions and experimental data for a *pin2* mutant. (Supports Figure 2.)
(A) Confocal image of an *pin2-DII-VENUS* root tip, reporting DII-VENUS (yellow) and propidium iodide staining (red).
(B) Measured DII-VENUS levels extracted from the confocal image shown in (A).
(C-G) Predicted auxin concentrations for each of the five ABCB-PIN interaction scenarios.
(H-L) Predicted auxin fluxes for each of the five ABCB-PIN interaction scenarios.
(M-Q) Predicted DII-VENUS concentrations for each of the five ABCB-PIN interaction scenarios.
(R-V) Mean DII-VENUS in the meristematic and elongation-zone regions of the epidermis and cortex, comparing the model predictions in each of the five ABCB-PIN interaction scenarios with the experimental data. Error bars show ±1 s.e.m.

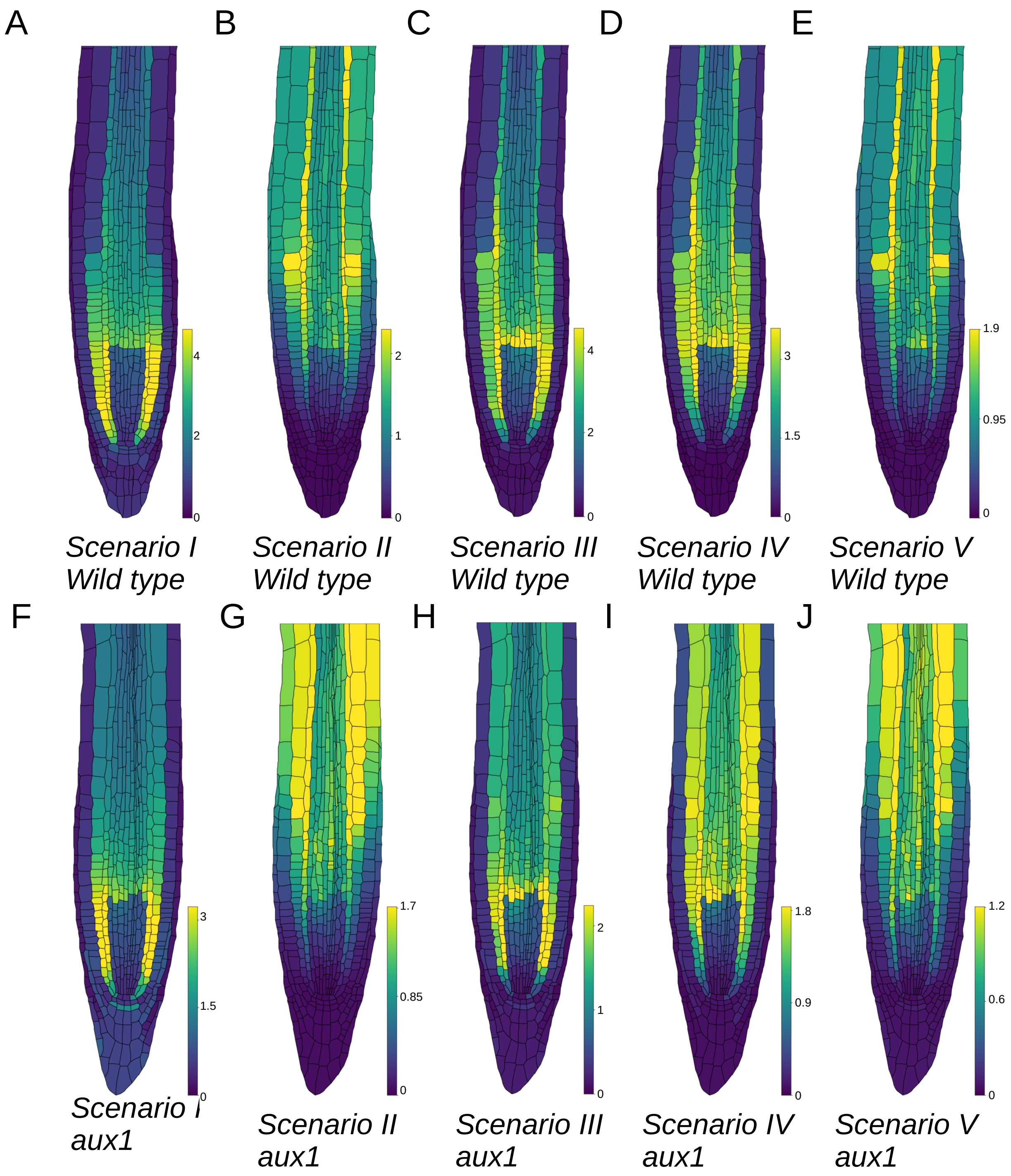

Supplemental Figure 7: Model predictions with ABCB4 acting as an influx transporter.
(Supports Figure 2.)
(A-E) Predicted DII-VENUS in wild type for each of the five ABCB-PIN interaction scenarios.
(F-J) Predicted DII-VENUS in an *aux1* mutant for each of the five ABCB-PIN interaction scenarios.

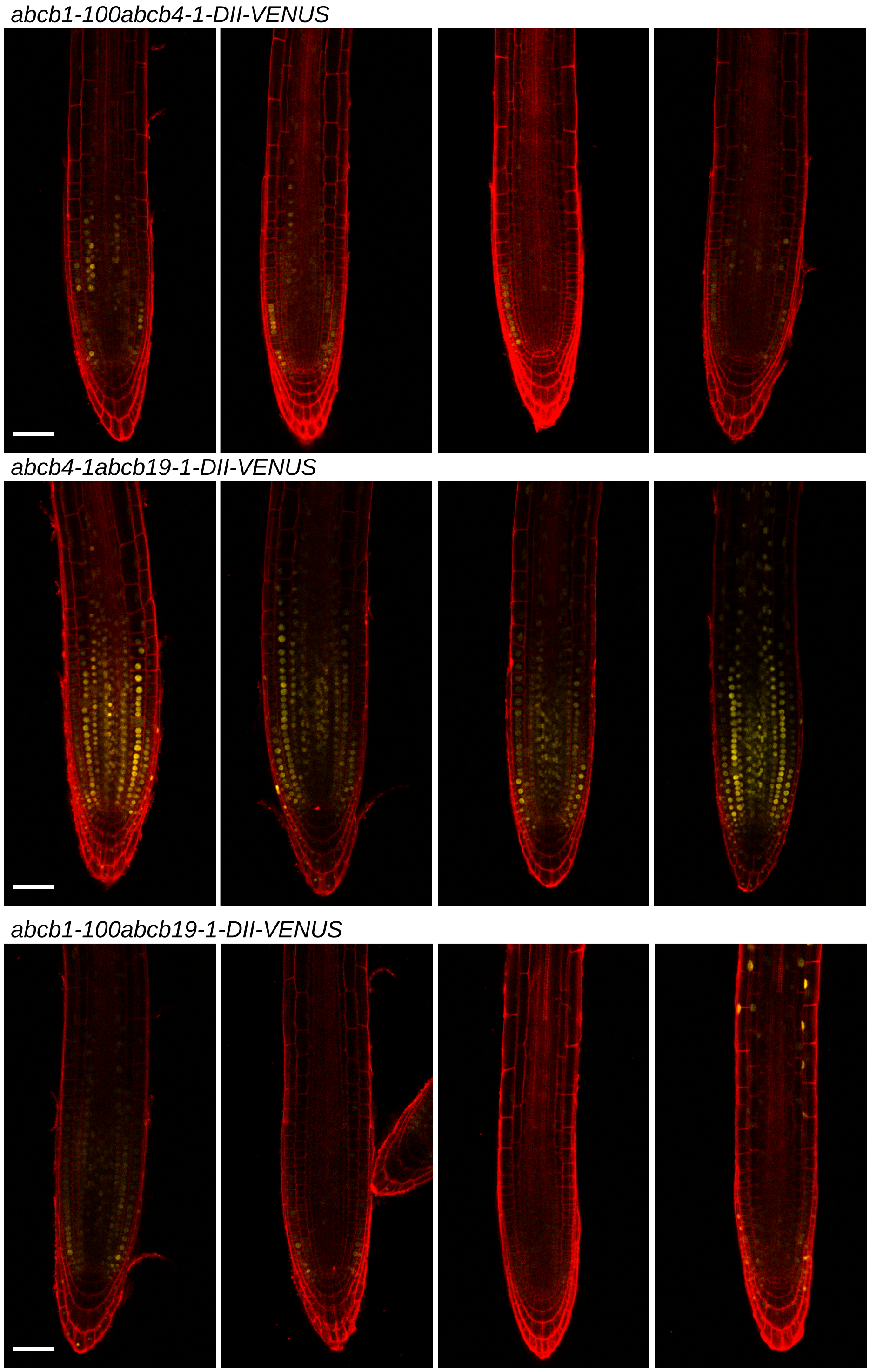

Supplemental Figure 8: Replicate DII-VENUS images for double mutant alleles *abcb1-100 abcb4-1*, *abcb4-1 abcb19-1* and *abcb1-100 abcb19-1*.
(Supports Figures 3 and 4.)

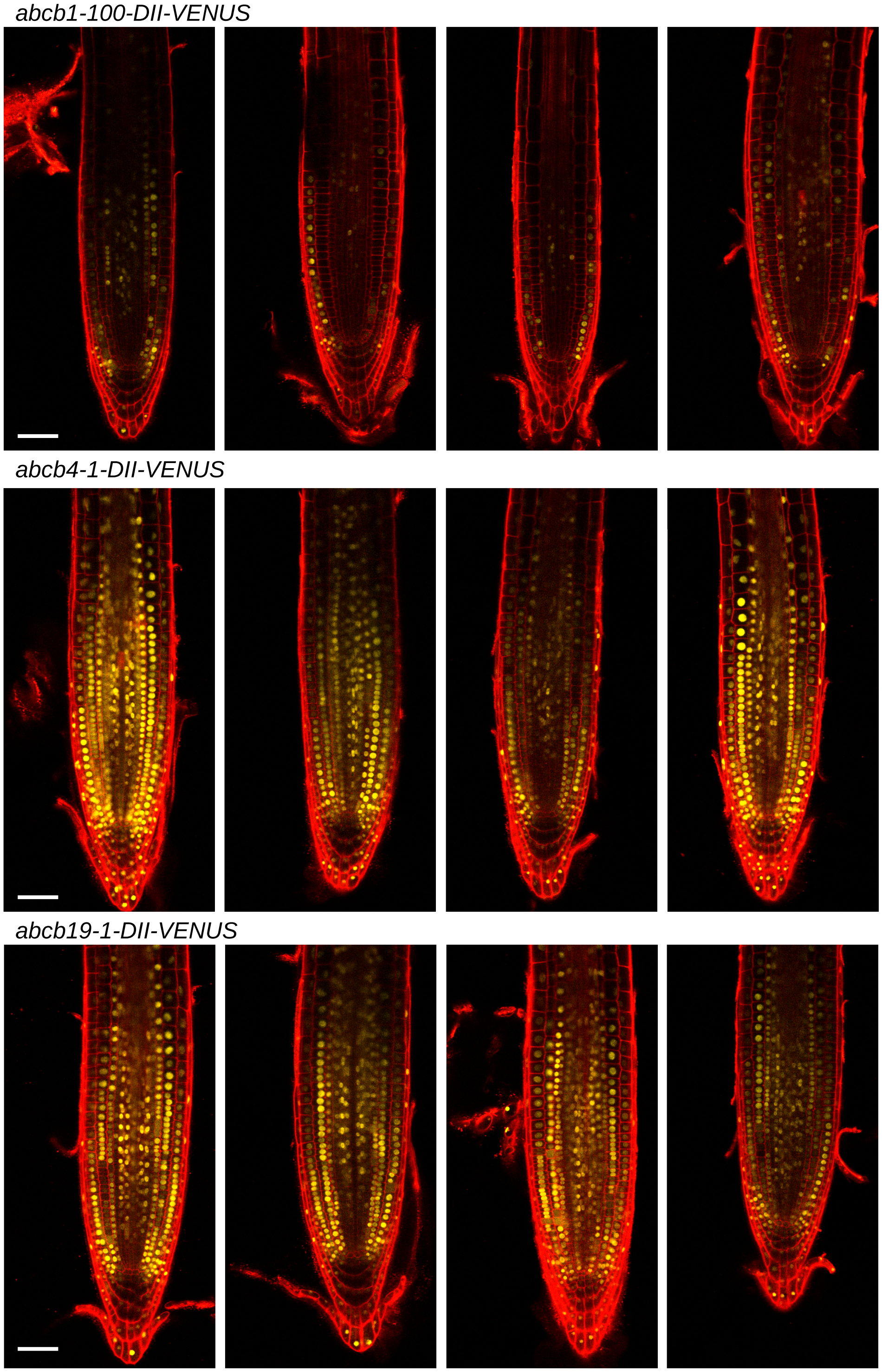

Supplemental Figure 9: Replicate DII-VENUS images for single mutant alleles *abcb1-100*, *abcb4-1* and *abcb19-1*. (Supports Figure 4.)

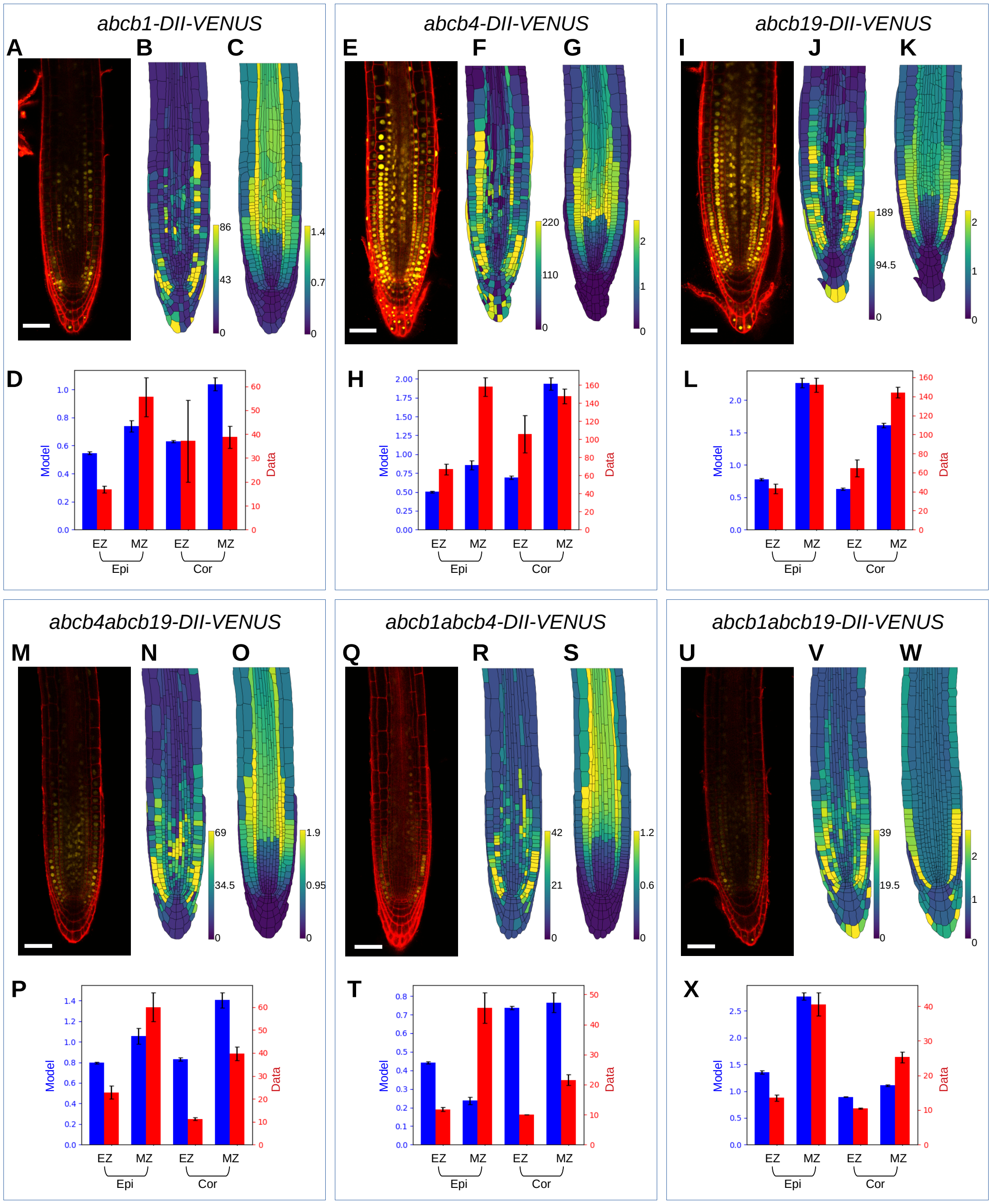

Supplemental Figure 10: Model predictions using scenario IV for the *abcb* single and double mutants assuming no upregulation of the remaining ABCBs.
(Supports Figure 4.)
(A-D) *abcb1*, (E-H) *abcb4*, (I-L) *abcb19*, (M-P) *abcb4 abcb19*, (Q-T) *abcb1 abcb4* and (U-X) *abcb1 abcb19*.
(A,E,I,M,Q,U) Confocal image with DII-VENUS (yellow) with propidium iodide staining (red).
(B,G,J,N,R,V) Measured DII-VENUS levels extracted from the corresponding confocal images.
(C,G,K,O,S,W) Predicted DII-VENUS distributions.
(D,H,L,P,T,X) Mean DII-VENUS in the meristematic and elongation-zone regions of the epidermis and cortex, comparing the model predictions and experimental data. Error bars show ±1 s.e.m.
To aid comparisons, the experimental data presented here is a repeat of that in Figure 4.

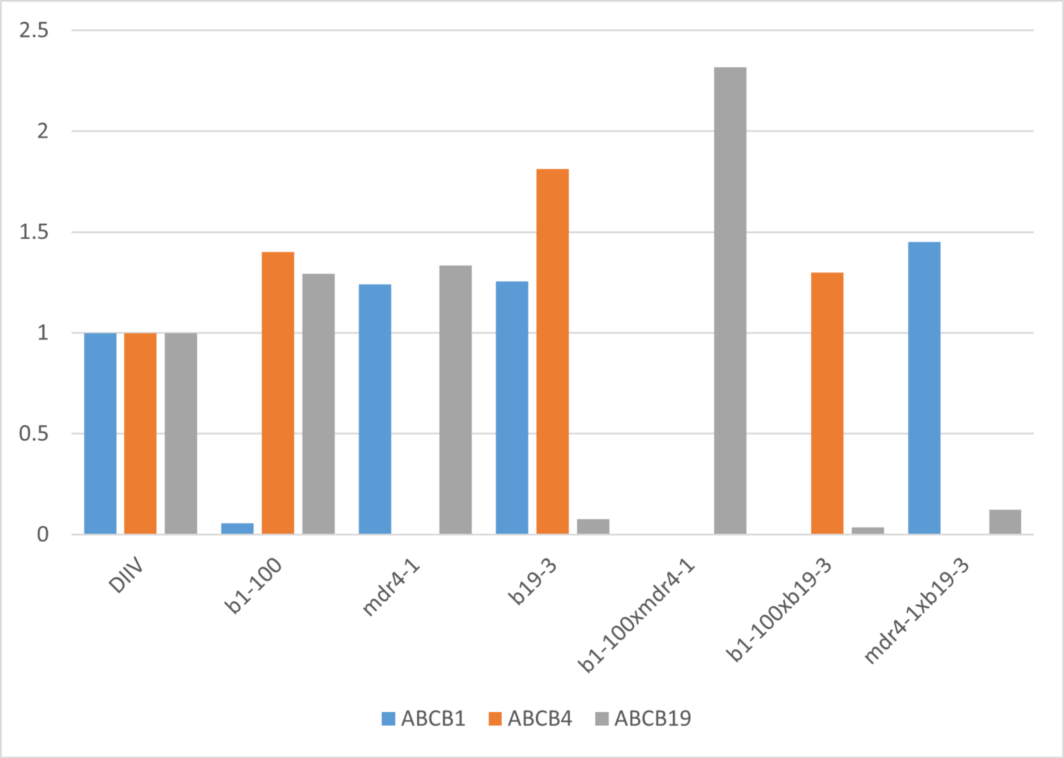

Supplemental Figure 11: Experimental RT-qPCR measurements of the relative expression of ABCB1, ABCB4 and ABCB19 in the *abcb* single and double mutants.
(Supports Figure 4.)
Measurements are given relative to DII-VENUS wild type (DIIV) for alleles b1-100 (*abcb1*), mdr4-1 (*abcb4*), b19-3 (*abcb19*), b1-100×mdr4-1 (*abcb1 abcb4*), b1-100×b19-3 (*abcb1 abcb19*) and mdr4-1×b19-3 (*abcb4 abcb19*).

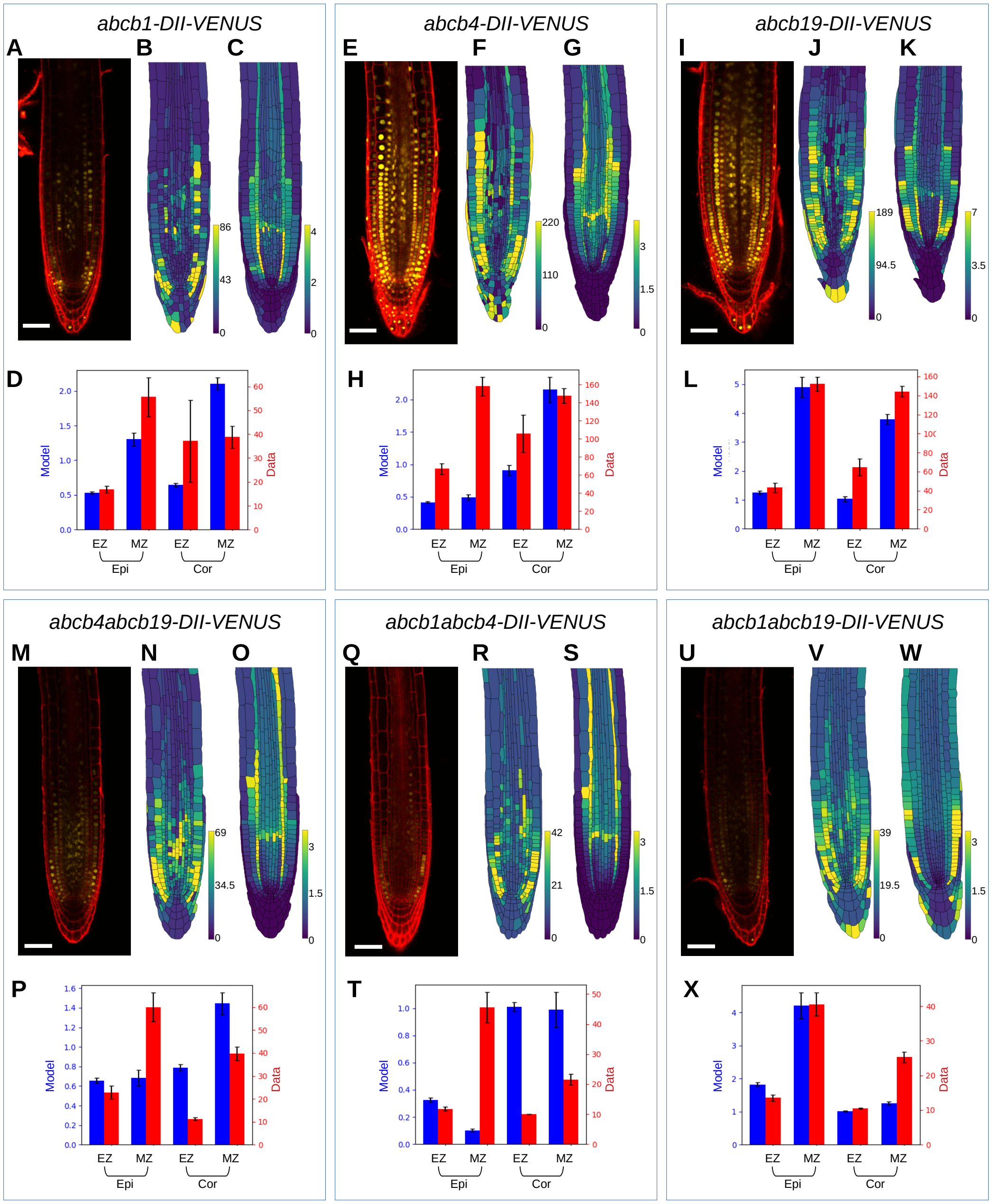

Supplemental Figure 12: Model predictions using scenario III for the *abcb* single and double mutants assuming a large synergistic efflux ($P_{SYN} = 3.0\,\mu$m/s)
(Supports Figure 4.)
(A-D) *abcb1*, (E-H) *abcb4*, (I-L) *abcb19*, (M-P) *abcb4 abcb19*, (Q-T) *abcb1 abcb4* and (U-X) *abcb1 abcb19*.
(A,E,I,M,Q,U) Confocal image with DII-VENUS (yellow) with propidium iodide staining (red).
(B,G,J,N,R,V) Measured DII-VENUS levels extracted from the corresponding confocal images.
(C,G,K,O,S,W) Predicted DII-VENUS distributions.
(D,H,L,P,T,X) Mean DII-VENUS in the meristematic and elongation-zone regions of the epidermis and cortex, comparing the model predictions and experimental data. Error bars show ±1 s.e.m.
To aid comparisons, the experimental data presented here is a repeat of that in Figure 4.

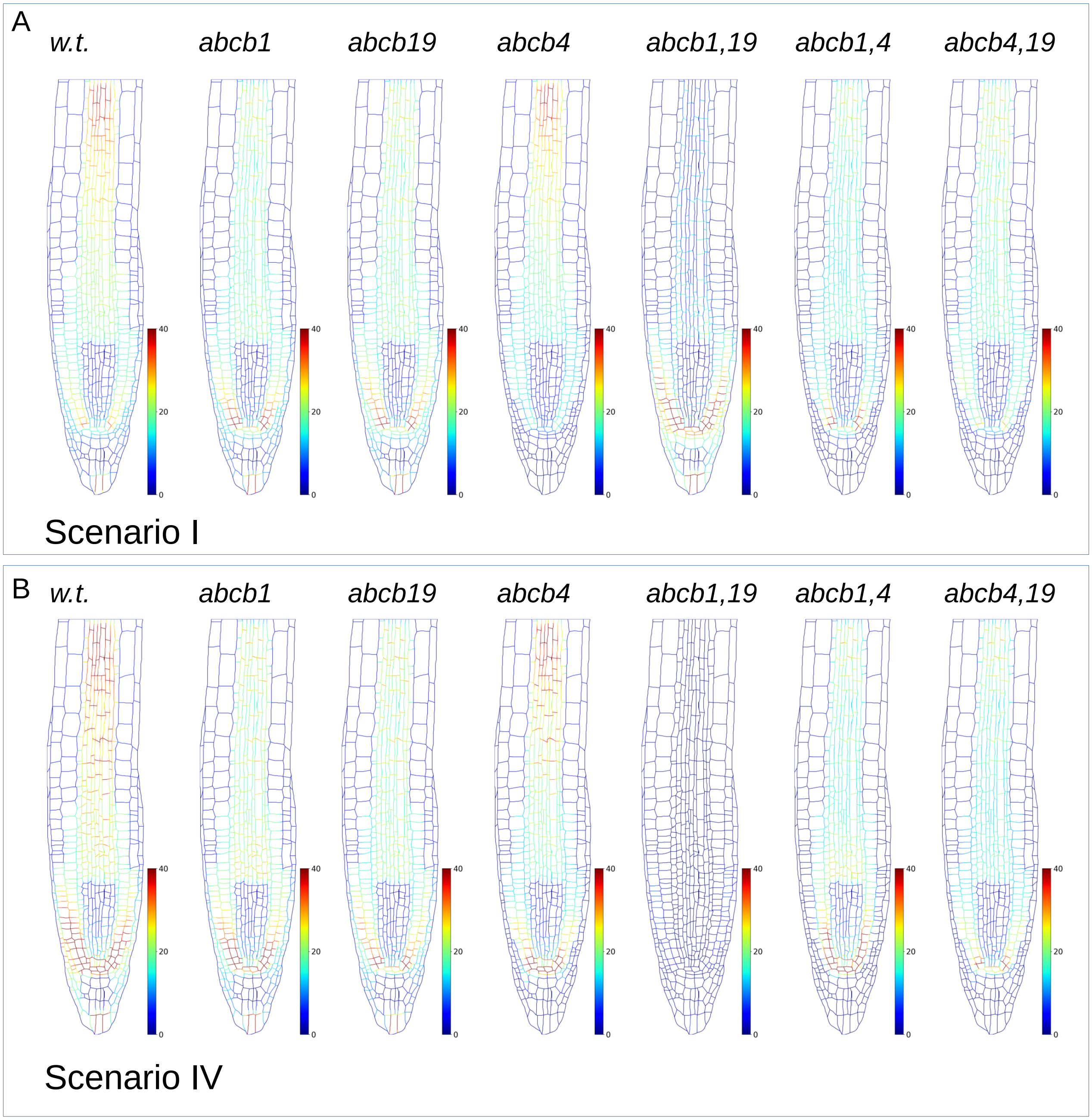

Supplemental Figure 13: Predicted apoplastic auxin concentrations.
(Supports Figures 2 and 4.)
(A) ABCB-PIN interaction scenario I. (B) ABCB-PIN interaction scenario IV.
Predictions show that amount of apoplastic auxin is reduced in the *abcb* single and double mutants. In the case of *abcb1 abcb19* with scenario IV, very little auxin is predicted to be in the apoplast.

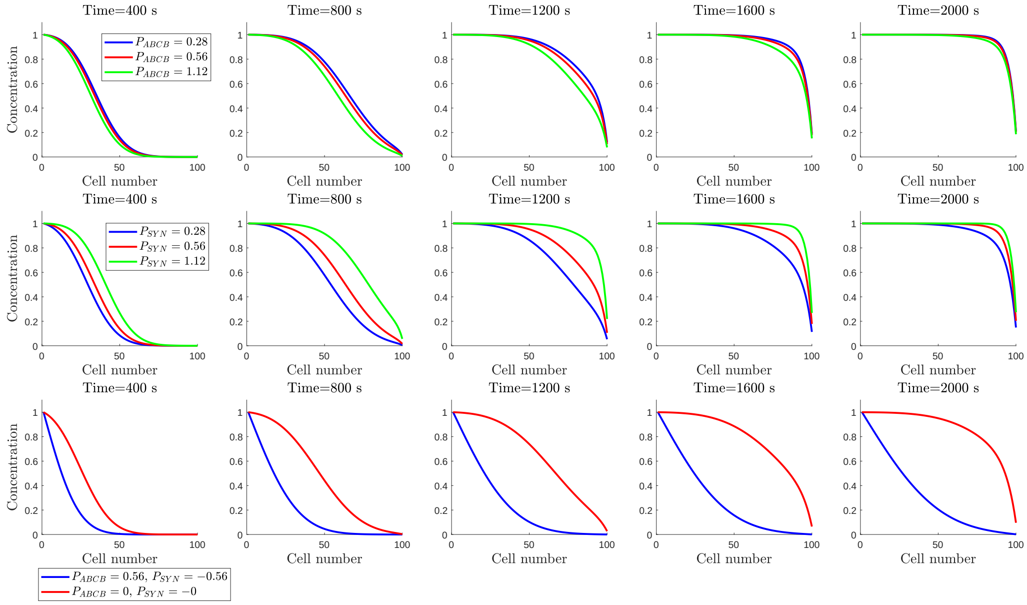

Supplemental Figure 14: Predicted auxin distributions for the single cell-file model showing the effect of the ABCB-independent and co-dependent permeability parameters.
(Supports Figure 5).
Each simulation includes independent PIN-mediated efflux with a permeability, $P_{PIN} = 0.56\mu\text{m}\,\text{s}^{-1}$.
Top row: the effect of different values for the rate of independent ABCB efflux (P_{ABCB}).
Middle row: the effect of different values for the rate of ABCB- PIN synergistic efflux (P_{SYN}).
Bottom row: the effect of an antagonistic co-dependent ABCB-PIN mediated efflux. Here, the red line corresponds to no ABCB-mediated efflux ($P_{ABCB} = P_{SYN} = 0$), whereas the blue line corresponds to having both an independent ABCB-mediated efflux and an antagonistic co-dependent efflux where both ABCB and PIN are present.

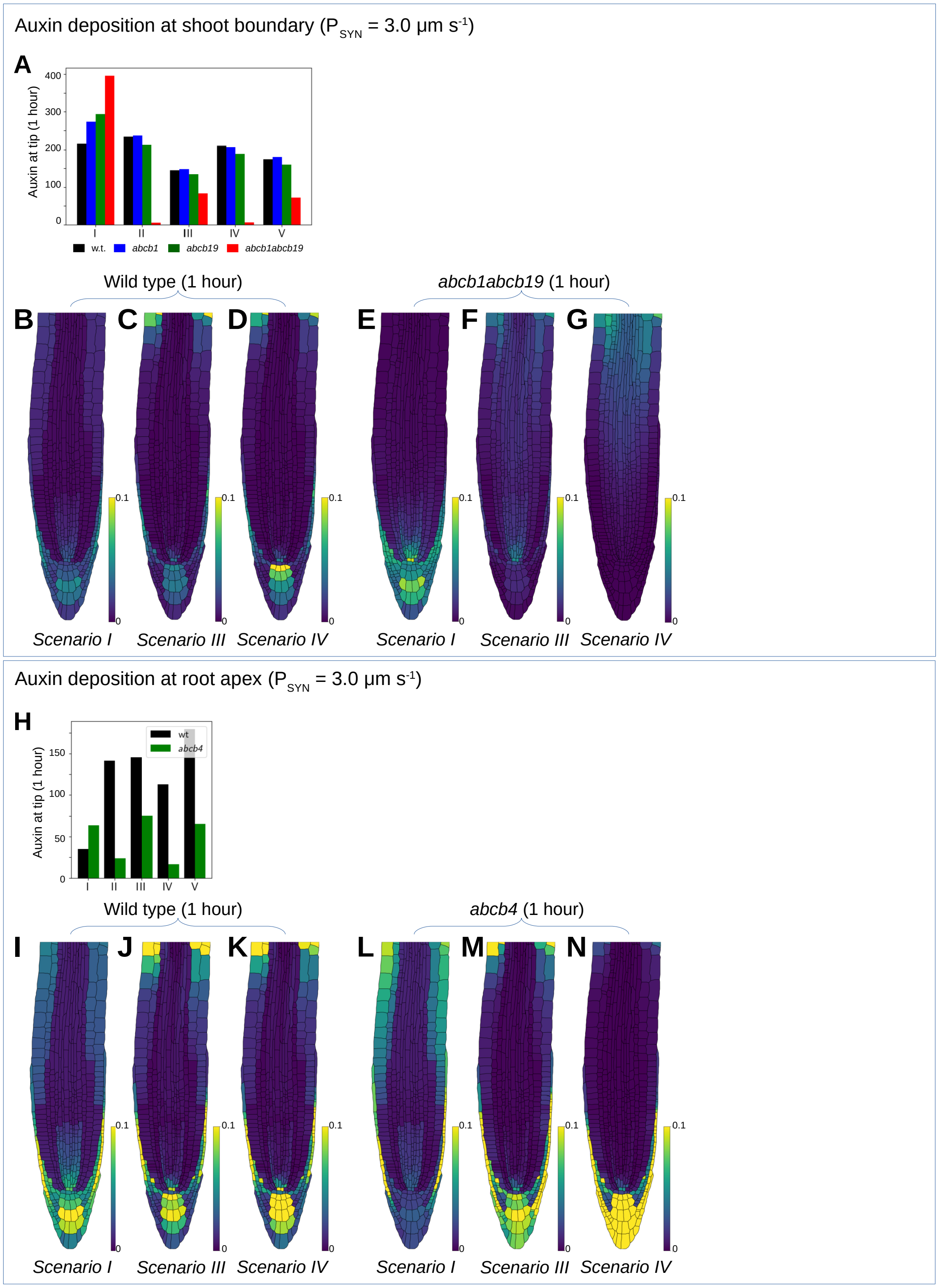

Supplemental Figure 15: Predicted long-distance auxin transport assuming a large synergistic efflux ($P_{SYN} = 3.0\,\mu\mathrm{m/s}$).
(Supports Figure 6.)
(A-G) Auxin distributions one hour after deposition at the shoot boundary.
(A) Total predicted auxin at the root apex for each of the five scenarios for wild type, *abcb1*, *abcb19* and *abcb1 abcb19*.
(B-D) Predicted wild-type auxin distribution for scenarios I (B), III (C) and IV (D).
(E-G) Predicted auxin distribution in *abcb1 abcb19* for scenarios I (E), III (F) and IV (G).
(H-N) Auxin distributions one hour after deposition at the root tip.
(H) Total predicted auxin at the shootward boundary for each of the five scenarios for wild type and *abcb4*.
(I-K) Predicted wild-type auxin distribution for scenarios I (I), III (J) and IV (K).
(L-N) Predicted auxin distribution in *abcb4* for scenarios I (L), III (M) and IV (N).

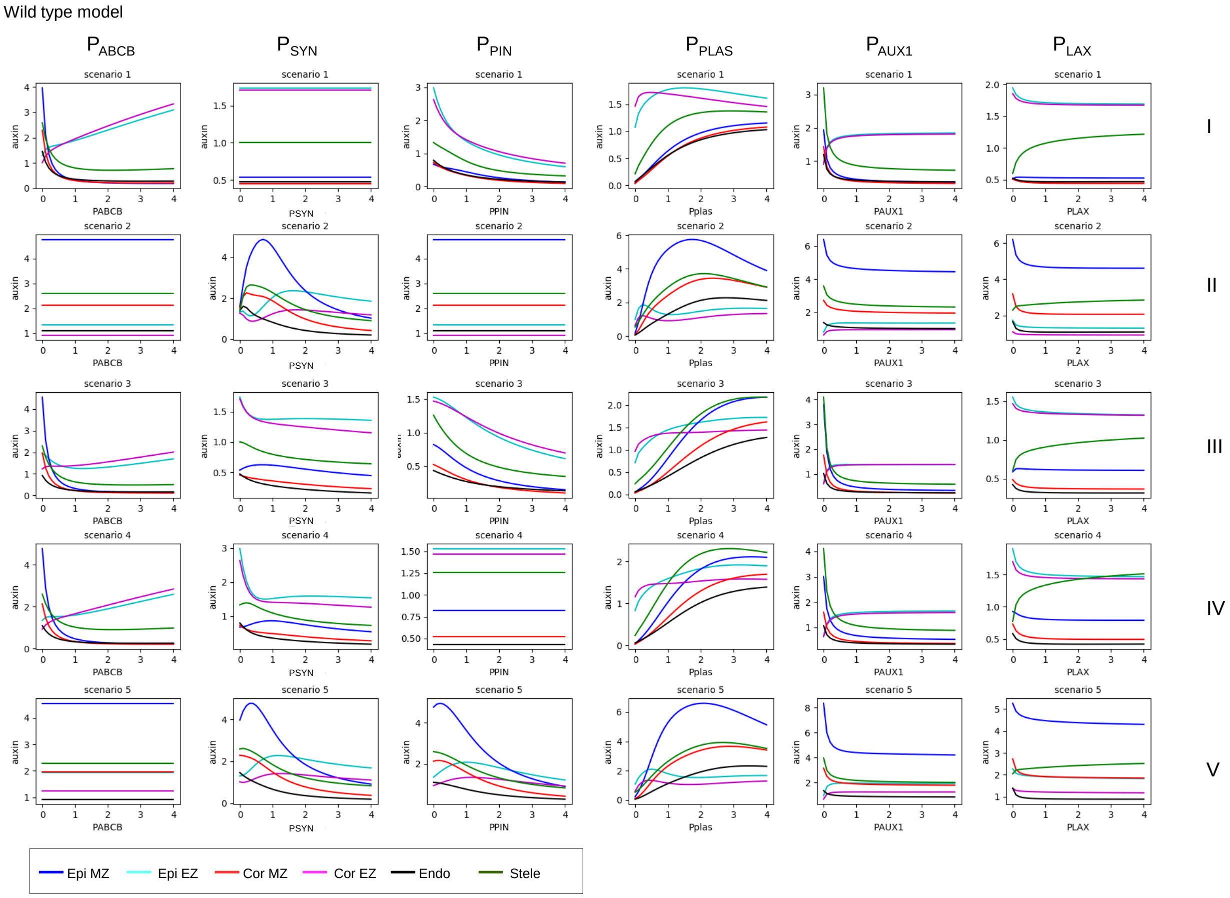

Supplemental Figure 16: Influence of the permeability parameters on the predicted mean auxin concentration in specific tissues in wild type.
(Supports Figure 2).

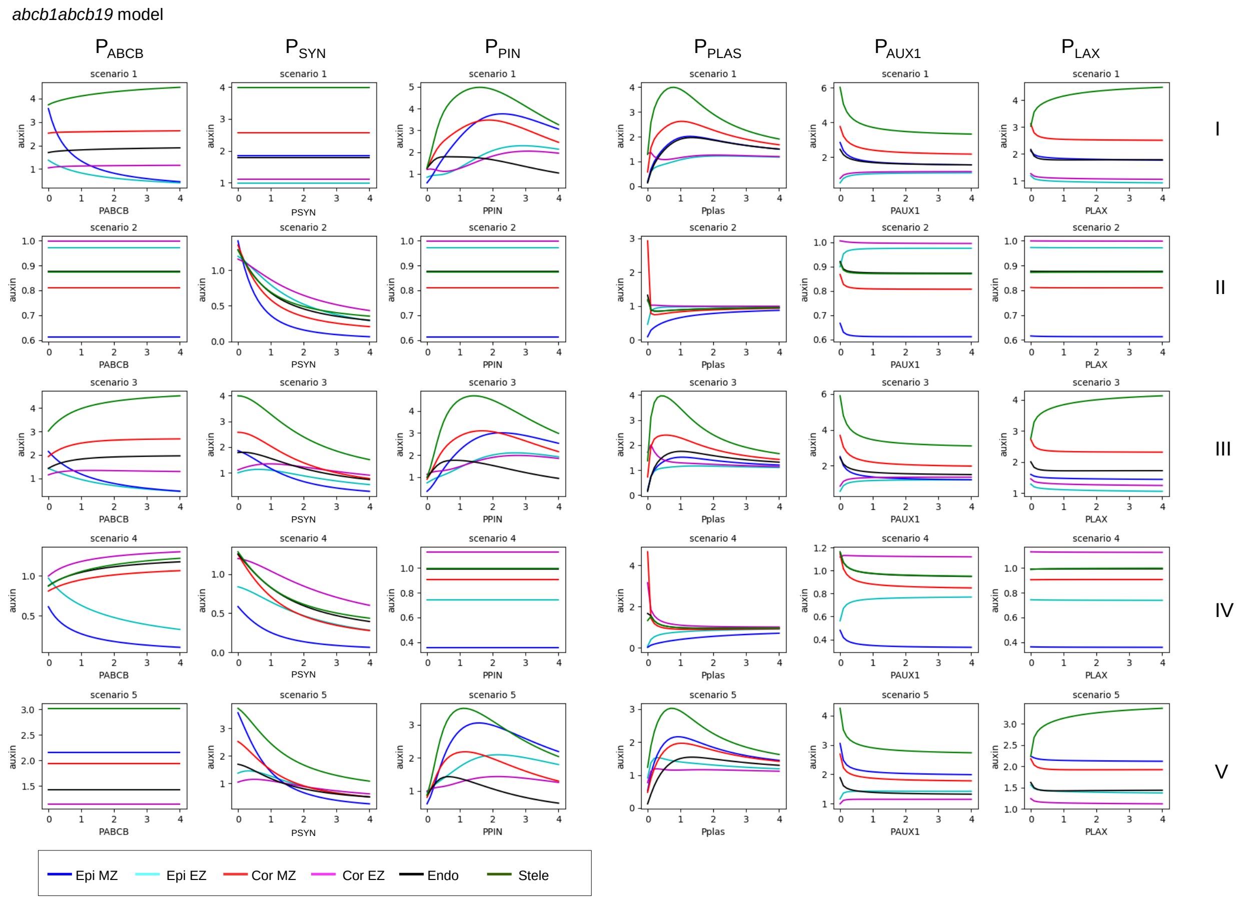

Supplemental Figure 17: Influence of the permeability parameters on the predicted mean auxin concentration in specific tissues in the *abcb1 abcb19* mutant.
(Supports Figure 3).

Supplemental Method. Detailed model description.

The computational model simulates auxin dynamics with a multicellular root tip geometry. The modelling framework was first described in (2). The new model presented here uses the same assumptions as that in (9), except that instead of a uniform background efflux term 'P_{back}', we prescribe the spatial distribution of three ABCB efflux transporters ABCB1, ABCB4 and ABCB19, and model auxin efflux mediated by these ABCBs (see below for further details). In this supplementary material, we summarise the modelling framework and model equations, providing a detailed description of the new ABCB model and corresponding equations.

S1 Characterising the multicellular root geometries and carrier distributions

S1.1 Defining the real multicellular root geometries

As in (2) (Supplemental methods, section 1) the root geometries are based on a 2-dimensional multicellular structure of a cross-secion of the *Arabidopsis* root apex (approximately 500μm shootwards from the root tip) obtained using confocal microscopy images in which cell walls were stained with propidium iodide (see Materials and Methods). In each case, the roots expressed the DII-VENUS nuclear-located yellow-fluorescent-protein auxin sensor (3; 4). The software package SurfaceProject ((2), supplemental methods, section 1.1) was used to extract a 2D plane from each image stack with all the nuclei brought into the plane of focus.

Once the 2D image was obtained, the cell segmentation software CellSet (10) was used to extract the position of all cell walls, and quantify DII-VENUS nuclear fluorescence for every cell. Cell types were input manually using CellSet, according to the template shown in Figure S2.

The tissue is set up using Python code based on the Openalea framework (11), and defined as a set of points in space representing the vertices of the multicellular geometry, which are linked to wall objects, which in turn are linked to objects representing the cell compartments. Wall compartments may be shared between two cells in the tissue (which we define as 'inner' walls), or if associated with a cell at the edge of the tissue it is only associated with that one cell (and defined as an 'outer' wall). Cell membranes are represented by way of a directed graph between the cells.

The 2D area of a given apoplast compartment is equal to the length of the associated cell wall multiplied by the cell-wall thickness, λ. The area of the small vertex compartments are approximated as λ^2. Finally, the area of a given cell is calculated as the area of the irregular polygon defined by the vertices bounding the walls adjacent to that cell, which can be calculated using a triangulation algorithm.

S1.2 Defining the geometrically regular multicellular root geometry

The geometrically regular root geometry used in Figure S3 is made by repeated subdivisions of an outline template created using Openalea and based on the mean values of cell measurements taken from confocal images of the root tip (summarised in Table S1). Using these values, the outline and subdivisions were made using Python and Openalea.

S1.3 Specifying the distributions of the PIN, AUX1/LAX and ABCBs

PIN1,2,3,4 and 7 efflux carriers and AUX1, LAX2 and LAX3 influx carriers are positioned on cell membranes according to a rules based on cell type, position and membrane orientation, as described in (2), Supplemental Tables 2 and 3. These carrier distributions are summarised in Fig S2D-K. The ABCB transporters introduced in this paper are specified on the cell membranes following the distributions shown in Fig 1D, S2A-C (based on the GFP images shown in Fig 1A-C, as described in the main text).

To simulate the auxin dynamics in the *abcb*, *pin2* and *aux1* mutants, we set the corresponding carrier value to zero on every membrane.

S2 Model equations for auxin

The model comprises a system of coupled ordinary differential equations (ODEs) for the auxin concentration within each cell and each apoplast compartment (assuming that the auxin concentration is homogeneous within each of these compartments). These ODEs contain terms representing passive diffusion of protonated auxin across cell membranes (§S2.1), carrier-mediated fluxes across cell membranes (see §S2.2 and §S2.3), diffusion within the apoplast (see §S2.4), cell-to-cell diffusion through plasmodesmata (see §S2.5) and auxin synthesis and degradation (see §S2.6). Below, we describe each of these components in turn, before describing the coupled system of ODEs in §S2.7.

S2.1 Passive diffusion of protonated auxin across cell membranes

Protonated auxin can passively diffuse across cell membranes. We label the apoplast compartments between cells i and j by ijk for $k = 1, 2, \ldots, Nij$. Then following (2), the passive flux of protonated auxin from apoplast compartment ijk to cell i is given by

$$\mathrm{J}_{\mathrm{ijk}}^{\mathrm{pass}} = P_{IAAH}(A_1[\mathrm{Auxin}]_{aijk} - B_1[\mathrm{Auxin}]_i), \tag{1}$$

where $[\mathrm{Auxin}]_i$ is the auxin concentration in cell i, and $[\mathrm{Auxin}]_{aijk}$ the auxin concentration in apoplastic compartment ijk, A_1 is the proportion of protonated auxin in the apoplast, B_1 is the proportion of protonated auxin in the cell cytoplasm, and P_{IAAH} is the permeability associated with this passive diffusion. The proportion of protonated auxin in each compartment, A_1 and B_1, depends on the compartmental pH, as described in (1) and given in Table S4.

S2.2 Carrier-mediated flux via AUX1/LAX and PINs (independent of ABCBs)

The AUX1/LAX and PIN carriers can mediate influx and efflux of anionic auxin across cell membranes respectively. In this subsection, we define these carrier-mediated flux, considering here only the PIN efflux component that is independent of the ABCBs, before introducing the ABCB-dependent efflux in the next subsection.

The AUX1/LAX and PIN mediated fluxes only occur for membranes where the corresponding carrier is present. Considering membrane segment k on the membrane of cell i which faces cell j, we introduce $[\mathrm{AUX1}]_{ijk}$ which is equal to one if AUX1 is present or zero otherwise, $[\mathrm{LAX}]_{ijk}$ which is equal to one if LAX2 or LAX3 are present and zero otherwise, and $[\mathrm{PIN}]_{ijk}$ which is equal to the number of members of the PIN family present on the membrane. As in (2), we can calculate the flux mediated by the carriers from apoplastic compartment ijk into cell i via:

$$\begin{aligned}\mathrm{J}^{\mathrm{carr}}_{\mathrm{ijk}} =& P_{AUX1}[\mathrm{AUX1}]_{ijk}(A_2[\mathrm{Auxin}]_{aijk} - B_2[\mathrm{Auxin}]_i)\\ &+ P_{LAX}[\mathrm{LAX}]_{ijk}(A_2[\mathrm{Auxin}]_{aijk} - B_2[\mathrm{Auxin}]_i)\\ &+ P_{PIN}[\mathrm{PIN}]_{ijk}(A_3[\mathrm{Auxin}]_{aijk} - B_3[\mathrm{Auxin}]_i),\end{aligned} \tag{2}$$

where A_2, A_3, B_2 and B_3 are constants (described in (1) and given in Table S4), P_{PIN}, P_{AUX1} and P_{LAX} are the permeabilities associated with each flux component (see Tables S3 and S4), $[\mathrm{Auxin}]_i$ is the auxin concentration in cell i, and $[\mathrm{Auxin}]_{aijk}$ the auxin concentration in apoplastic compartment ijk.

S2.3 ABCB-mediated efflux

As described in the main text, we consider five possible ABCB-PIN interaction scenarios:

(I) PIN and ABCB act independently,

(II) PIN and ABCB are entirely co-dependent and both must be present for either to efflux auxin,

(III) PIN and ABCB act independently, but where both are present on a given membrane there is an additional synergistic efflux,

(IV) ABCB efflux auxin independently; however, PINs are not able to efflux auxin in the absence of ABCBs but instead enable a co-dependent efflux where both ABCB and PIN are present,

(V) PIN efflux auxin independently; however, ABCBs are not able to efflux auxin in the absence of PINs but instead enable a co-dependent efflux where both ABCB and PIN are present.

Considering these five scenarios requires us to introduce two further flux terms: an ABCB-mediated efflux which is independent of PINs, and a co-dependent efflux that occurs where both ABCB and PIN are present. Denoting the permeability parameter for PIN-independent ABCB efflux by P_{ABCB}, and the permeability parameter for the

'synergistic' efflux where both PIN and ABCB are present by P_{SYN}, the two additional ABCB-mediated efflux components take the form:

$$\begin{aligned} \mathrm{J_{ijk}^{ABCB}} =& - B_3 P_{ABCB} \left([\mathrm{ABCB1}]_{ijk} + [\mathrm{ABCB4}]_{ijk} + [\mathrm{ABCB19}]_{ijk} \right) [\mathrm{Auxin}]_i \\ & - B_3 P_{SYN} \left([\mathrm{ABCB1}]_{ijk} + [\mathrm{ABCB4}]_{ijk} + [\mathrm{ABCB19}]_{ijk} \right) [\mathrm{PIN}]_{ijk} [\mathrm{Auxin}]_i \end{aligned} \quad (3)$$

where B_3 is a constant (see (1) and Table S4) and $[\mathrm{ABCB1}]_{ijk}$, $[\mathrm{ABCB4}]_{ijk}$, and [ABCB19] are one or zero depending on whether the respective ABCB is present on a given membrane. We note that unlike other carrier-mediated fluxes which act to move anionic auxin down potential gradients, ABCB activity is ATP-driven and so can act against any potential gradient.

In scenario (III), P_{ABCB}, P_{SYN} and P_{PIN} are all set to be non-zero; however, in the other scenarios one or more of these parameters are set to zero, effectively removing that component of the efflux. The values for each of these parameters in each case are given in Table S3.

S2.4 Auxin diffusion with the apoplast

To simulate auxin diffusion within the apoplast, the flux between adjacent apoplast compartments is simulated by considering the flux from each compartment to and from two small vertex compartments representing the two ends of a given apoplast compartment. Denoting the flux from vertex l to apoplast ijk as J_{ijkl} we have the following:

$$\mathrm{J_{ijkl}} = \frac{\mathrm{2D_{cw}}}{\mathrm{S_{ijk}}} ([\mathrm{Auxin}]_{\mathrm{vl}} - [\mathrm{Auxin}]_{\mathrm{aijk}}), \quad (4)$$

where D_{cw} is the diffusion coefficient of auxin in the apoplast, S_{ijk} the length of compartment ijk, and $[\mathrm{Auxin}]_{vl}$ the auxin concentration in vertex l.

S2.5 Auxin diffusion through plasmodesmata

As in (9), we also include auxin diffusion between adjacent cell cytoplasms via intercellular plasmodesmata. The plasmodesmatal auxin flux from cell j to cell i across apoplast compartment ijk, is therefore defined as:

$$\mathrm{J_{ijk}^{plas}} = \mathrm{P_{plas}}\ \mathrm{d_{ij}}([\mathrm{Auxin}]_{\mathrm{j}} - [\mathrm{Auxin}]_{\mathrm{i}}) \quad (5)$$

where P_{plas} is the permeability per plasmodesmata ($\mu m^3 s^{-1}$) and d_{ij} the plasmodesmatal density (μm^{-2}) between cells i and j ($d_{ij} \equiv d_{ji}$). The plasmodesmatal density varies by cell type and cell-wall orientation and is specified using TEM data given by (16).

S2.6 Auxin synthesis and degradation

We also include auxin synthesis and degradation. As described in the main text and following (9), we prescribe a high rate of auxin synthesis in the QC, initials and outer lateral root cap, $\alpha_i = \alpha_i^{HIGH}$, and a low rate of auxin synthesis in all other cells, $\alpha_i = \alpha_i^{LOW}$. We prescribe a uniform degradation rate, β.

S2.7 Coupled system of ODEs for auxin concentrations

Combining the fluxes given by Equations (1)-(5) and the synthesis and degradation terms, we obtain the following system of coupled ODEs for the auxin concentrations:

$$\frac{\mathrm{d[Auxin]}_i}{\mathrm{d}t} = \alpha_i - \beta[\mathrm{Auxin}]_\mathrm{i} + \frac{1}{\mathrm{R_i}}\sum_{\mathrm{j}\in\mathrm{C_i}}\sum_{\mathrm{k}=1}^{\mathrm{N_{ij}}}\mathrm{S_{ijk}}\left(\mathrm{J^{pass}_{ijk}} + \mathrm{J^{carr}_{ijk}} + \mathrm{J^{ABCB}_{ijk}} + \mathrm{J^{plas}_{ijk}}\right), \quad (6)$$

$$\frac{\mathrm{d[Auxin]}_{aijk}}{\mathrm{d}t} = -\frac{1}{\lambda}(\mathrm{J_{ijk}} + \mathrm{J_{jik}}) + \frac{1}{\mathrm{S_{ijk}}}\sum_{\mathrm{m}\in\mathrm{V_{ijk}}}\mathrm{J_{ijkm}}, \quad (7)$$

$$\frac{\mathrm{d[Auxin]}_{vk}}{\mathrm{d}t} = -\frac{1}{\lambda}\sum_{\mathrm{ijk}\in\mathrm{W_l}}\mathrm{J_{ijkl}}, \quad (8)$$

where $\mathrm{R_i}$ is the 2-D area of cell i, C_i denotes the set of cells adjacent to cell i, N_{ij} denotes the number of apoplast compartments between cells i and j, V_{ijk} denotes the pair of vertex compartments adjacent to apoplast compartment ijk and W_l denotes the set of apoplast compartments, ijk, adjacent to vertex l.

S3 Numerical methods

Where we present steady-state auxin distributions, we compuate the steady state directly as follows. Given the system is linear, we can compute the steady state by setting the derivatives to zero and rearranging the system into the form:

$$\mathbf{J} \times [\mathbf{Auxin}] = \mathbf{r},$$

where (if n is the total number of cell, apoplast and vertex compartments) $\mathbf{J}$ is the $n \times n$ matrix representing all of the combined fluxes and degradation terms, $[\mathbf{Auxin}]$ is the $n \times 1$ vector of auxin concentrations in every compartment, and $\mathbf{r}$ the $n \times 1$ vector of production rates and boundary conditions. The resulting linear system is then solved using the sparse matrix solver spsolve from the Python package Numpy.

In cases where we present dynamic auxin distributions (i.e. where we investigate long-distance auxin transport), we compute the time-dependent solution using a sparse ODE solver based on Scipy odeint.

S3.1 Boundary and initial conditions

For cells in the stele at the shootward boundary we assume a constant fixed supply of auxin from the shoot so there is a fixed boundary condition of auxin ($[\mathrm{Auxin}]_\mathrm{b} = 1$) in these cells. For the remaining cells at the shootward boundary (i.e. pericycle, endodermis, cortex and epidermis), we follow (8; 9) in assuming a zero gradient boundary condition, so that at steady state, the auxin concentration in these cells is equal to the value in the adjacent cell in the same cell layer; i.e. for a given outer boundary cell o with rootward neighbour n we have, at steady state:

$$[\mathrm{Auxin}]_o - [\mathrm{Auxin}]_n = 0$$

In the simulations of long-distance auxin transport, we present dynamic auxin distributions and therefore also require an initial condition. We consider auxin deposition at

either the shoot boundary or the root apex, as shown in Figure 6, and therefore prescribe initial conditions with auxin concentration equal to zero everywhere except in the subset of cells indicated in Figure 6A (shoot boundary) or 6C (root apex), where auxin concentration is set to one. The full ODE system is then solved for one hour model time for each of the various scenarios and mutant cases shown.

S4 Model equations for DII-VENUS

As in (9), we first calculate the steady-state auxin distribution (as described above) and then use this solution to calculate the steady-state DII-VENUS distribution. The following equations are identical to those used previously in (9), however are provided here for completeness.

Following (3), the DII-VENUS dynamics within each cell can be described using a system of coupled nonlinear ordinary differential equations (ODEs) for the concentrations of auxin, $[\text{Auxin}]_i$, DII-VENUS, $[\text{VENUS}]_i$, TIR1/AFB receptors, $[\text{TIR1}]_i$, auxin-TIR1/AFB complexes, $[\text{Auxin} \cdot \text{TIR1}]_i$, and auxin-TIR1/AFB-DII-VENUS complexes, $[\text{Auxin} \cdot \text{TIR1} \cdot \text{VENUS}]_i$:

$$\begin{aligned}
\frac{\mathrm{d}[\text{Auxin}]_i}{\mathrm{d}t} &= k_d[\text{Auxin} \cdot \text{TIR1}]_i - k_a[\text{Auxin}]_i[\text{TIR1}]_i + \alpha_i - \beta[\text{Auxin}]_i \\
&\qquad + \frac{1}{R_i} \sum_{j \in C_i} \sum_{k=1}^{N_{ij}} S_{ijk} \left(J_{ijk} + J_{ij}^{p} \right), \qquad (9) \\
\frac{\mathrm{d}[\text{TIR1}]_i}{\mathrm{d}t} &= -k_a[\text{Auxin}]_i \, [\text{TIR1}]_i + k_d[\text{Auxin} \cdot \text{TIR1}]_i, \qquad (10) \\
\frac{\mathrm{d}[\text{Auxin} \cdot \text{TIR1}]_i}{\mathrm{d}t} &= k_a[\text{Auxin}]_i \, [\text{TIR1}]_i - k_d[\text{Auxin} \cdot \text{TIR1}]_i \\
&\quad + (l_d + l_m)[\text{Auxin} \cdot \text{TIR1} \cdot \text{VENUS}]_i \\
&\quad - l_a[\text{Auxin} \cdot \text{TIR1}]_i \, [\text{VENUS}]_i, \qquad (11) \\
\frac{\mathrm{d}[\text{Auxin} \cdot \text{TIR1} \cdot \text{VENUS}]_i}{\mathrm{d}t} &= l_a[\text{Auxin} \cdot \text{TIR1}]_i \, [\text{VENUS}]_i \\
&\quad - (l_d + l_m)[\text{Auxin} \cdot \text{TIR1} \cdot \text{VENUS}]_i, \qquad (12) \\
\frac{\mathrm{d}[\text{VENUS}]_i}{\mathrm{d}t} &= \delta - l_a[\text{VENUS}]_i \, [\text{Auxin} \cdot \text{TIR1}]_i \\
&\qquad + l_d[\text{Auxin} \cdot \text{TIR1} \cdot \text{VENUS}]_i, \qquad (13)
\end{aligned}$$

where $i = 1, 2, \cdots, N$ labels the cells, and the final term in (9) represents the change in auxin concentration due to fluxes across the cell membrane as defined above.

As described in detail in (3) , if we scale these equations and suppose that complex formation occurs rapidly (i.e. the rate constants k_a, k_d, l_a, l_d and l_m are relatively large), we can reduce the network model to a single equation for the DII-VENUS concentration:

$$\frac{\mathrm{d}[\text{VENUS}]_i}{\mathrm{dt}} = p_2 \left(1 - \frac{[\text{Auxin}]_\text{i}[\text{VENUS}]_\text{i}}{p_3 + p_4[\text{Auxin}]_\text{i} + \text{p}_1[\text{Auxin}]_\text{i}[\text{VENUS}]_\text{i}} \right), \qquad (14)$$

where we define the parameters p_1, p_2, p_3 and p_4 as:

$$p_1 = \frac{[\text{Auxin} \cdot \text{TIR1} \cdot \text{VENUS}]_b}{[\text{TIR1}]_\text{T}}, \; p_2 = \delta/[\text{VENUS}]_b,$$

$$p_3 = \frac{[\text{TIR1}]_b}{[\text{TIR1}]_\text{T}}, \quad p_4 = \frac{[\text{Auxin} \cdot \text{TIR1}]_b}{[\text{TIR1}]_\text{T}},$$

and where the b subscript represents the steady-state value of a given variable at the stele shootward boundary (see section S3.1), and $[\text{TIR1}]_\text{T}$ is the total (conserved) concentration of TIR1/AFB receptors in each cell. We use the parameter values $p_3 = 0.91$, $p_4 = 0.03$ and $p_1 = 0.06$ as estimated in (3) and used in (2).

Given we calculate the steady-state auxin in every cell directly, we set the derivative of equation (14) to zero, and rearrange to obtain the following steady-state relationship:

$$[\text{VENUS}]^*_i = \frac{\frac{p_3}{[\text{Auxin}]^*_i} + p_4}{1 - p_1}, \tag{15}$$

where the $*$ superscript represents the steady-state values of Auxin and DII-VENUS in a given cell.

S5 Single-file simulations

To assess how ABCBs affect auxin fluxes and long-distance transport, we simulated auxin transport through a single file of cells, with PIN efflux carriers located on the downstream cell membranes and ABCB transporters on all membranes. The model incorporates passive diffusion of protonated auxin across cell membranes, PIN-mediated efflux of anionic auxin across cell membranes where PINs are present, ABCB-mediated auxin efflux on all membranes (i.e. ABCB-mediated efflux independent of PINs), synergistic auxin efflux where both PIN and ABCB are present, and plasmodesmatal diffusion of auxin between adjacent cell cytoplasms. Labelling the cells by $i = 1, \cdots, N$, we let $c_i(t)$ denote the auxin concentration in cell cytoplasm i and $f_i(t)$ denote the auxin concentration of the apoplast region neighbouring cell cytoplasms i and $i+1$, at time t. The auxin fluxes across each cell membrane are then given by

$$\begin{aligned} J_{cfi} =& (B_1 P_{IAAH} + B_3 P_{PIN} + (1 - B_1) P_{ABCB} + B_3 P_{SYN}) c_i \\ & - (A_1 P_{IAAH} + A_3 P_{PIN} + A_3 P_{SYN}) f_i \quad \text{for} \quad i = 1, 2, \cdots, N-1, \end{aligned} \tag{16}$$

$$J_{fci} = A_1 P_{IAAH} f_{i-1} - (B_1 P_{IAAH} + (1 - B_1) P_{ABCB}) c_i \quad \text{for} \quad i = 2, 3, \cdots, N, \tag{17}$$

where J_{cfi} denotes the flux from cell i to apoplast region i and J_{fci} denotes the flux from apoplast region $i-1$ to cell i. The auxin fluxes through plasmodesmata from cell i to cell $i+1$ are given by

$$J_i^{plas} = P_{plas}(c_i - c_{i+1}) \quad \text{for} \quad i = 1, 2, \cdots, N-1, \tag{18}$$

where P_{plas} denotes the permeability due to plasmodesmata.

The auxin dynamics are then governed by the following system of coupled ordinary differential equations (ODEs):

$$\frac{\mathrm{d}c_i}{\mathrm{d}t} = \frac{1}{l}(J_{fci} - J_{cfi} + J_{i-1}^{plas} - J_i^{plas}) \text{ for } i = 2, \cdots, N-1, \tag{19}$$

$$\frac{\mathrm{d}f_i}{\mathrm{d}t} = \frac{1}{\lambda}(J_{cfi} - J_{fc(i+1)}) \text{ for } i = 1, \cdots, N-1, \tag{20}$$

where l denotes the cell length and λ denotes the apoplast thickness.

These ODEs, (19), are simulated under the assumption that the concentration in cells $i = 1$ and $i = N$ are held fixed, $c_1(t) = 1$, $c_N(t) = 0$, and all other concentrations are initially zero, $c_i(0) = 0$ for $i = 2, 3, \cdots, N$, $f_i(0) = 0$ for $i = 1, 2, \cdots, N$. Figures 5B and S14 show the simulation results for parameter values $N = 100$, $l = 20\,\mu$m, $\lambda = 0.5\,\mu m$ and the remaining parameters equal to those given in Table S4. We use the model to investigate how the ABCB-PIN interaction scenarios affect the propagation of auxin through the tissue, which can be characterised by an auxin wave speed (i.e. the speed at which the mid-point of the auxin front traverses the tissue) (7).

S6 Evaluating the robustness of our conclusions to parameter estimates

To evalute whether our model predictions are robust to the choices of parameter estimates, we used a fitness function to evaluate to goodness of fit between model and data for wild type and the *abcb1abcb19* knockout mutant, focussing on the six permeability parameters, P_{ABCB}, P_{SYN}, P_{PIN}, P_{plas}, P_{AUX1} and P_{LAX}, since estimates of these are not well characterised in the literature (Figures S4, S16-S17). This fitness function is calculated as the sum of squared differences between mean DII-VENUS, normalised to peak values, in both model and data, in the four subsets of cells selected for comparison throughout the study (elongation zone epidermis, elongation zone cortex, meristematic epidermis and meristematic cortex) as these tissues have the greatest resolution in the experimental images.

For wild-type, we find that for wide ranges of the permeability parameter values the predicted DII-VENUS distributions are in better agreement with ABCB-PIN interaction scenarios I, III and IV than with II and V, i.e. better agreement when ABCB efflux auxin independently of PINs (Figures S4, S16). As one may expect from this conclusions, in these scenarios (I, III and IV), the agreement between the model predictions and data reduces as the permeability associated with the PIN-independent ABCB-mediated efflux reduces, P_{ABCB}, further demonstrating that the prescence of non-polar ABCB-mediated efflux (independent of PIN) is essential to create the auxin distribution.

Based on the results for wildtype, for the *abcb1abcb19* knockout mutant, we then focussed on the auxin distributions for ABCB-PIN interaction scenarios I, III and IV (the red, blue and black lines in Figure S4). We see that in agreement with the findings presented in the main text, for wide ranges of permeability parameter values the model predictions are in better agreement with the data under scenario IV than with scenarios I and III. However, with a large synergistic efflux rate, P_{SYN}, scenario III provides better agreement than scenario IV. With these parameter values the synergistic efflux is substantially larger than that mediated by the PINs alone, supporting the suggestion that PINs are predominantly functioning through a synergistic efflux with the ABCBs.

References

[1] L. R. Band and J. R. King. Multiscale modelling of auxin transport in the plant-root elongation zone. *J Math Biol*, 65(4):743–85, 2012.

[2] L. R. Band, D. M. Wells, J. A. Fozard, T. Ghetiu, A. P. French, M. P. Pound, M. H. Wilson, L. Yu, W. Li, H. I. Hijazi, J. Oh, S. P. Pearce, M. A. Perez-Amador, J. Yun,

E. Kramer, J. M. Alonso, C. Godin, T. Vernoux, T. C. Hodgman, T. P. Pridmore, R. Swarup, J. R. King, and M. J. Bennett. Systems analysis of auxin transport in the Arabidopsis root apex. *Plant Cell*, 26(3):862–75, 2014.

[3] L. R. Band, D. M. Wells, A. Larrieu, J. Sun, A. M. Middleton, A. P. French, G. Brunoud, E. M. Sato, M. H. Wilson, B. Péret, M. Oliva, R. Swarup, I. Sairanen, G. Parry, K. Ljung, T. Beeckman, J. M. Garibaldi, M. Estelle, M. R. Owen, K. Vissenberg, T. C. Hodgman, T. P. Pridmore, J. R. King, T. Vernoux, and M. J. Bennett. Root gravitropism is regulated by a transient lateral auxin gradient controlled by a tipping-point mechanism. *Proc Natl Acad Sci USA*, 109(12):4668–73, 2012.

[4] G. Brunoud, D. M. Wells, M. Oliva, A. Larrieu, V. Mirabet, A. H. Burrow, T. Beeckman, S. Kepinski, J. Traas, M. J. Bennett, and T. Vernoux. A novel sensor to map auxin response and distribution at high spatio-temporal resolution. *Nature*, 482(7383):103–6, 2012.

[5] R. J. Dyson, G. Vizcay-Barrena, L. R. Band, A. N. Fernandes, A. P. French, J. A. Fozard, T. C. Hodgman, K. Kenobi, T. P. Pridmore, M. Stout, D. M. Wells, M. H. Wilson, M. J. Bennett, and O. E. Jensen. Mechanical modelling quantifies the functional importance of outer tissue layers during root elongation and bending. *New Phytol*, 202(4):1212–22, 2014.

[6] E. M. Kramer, N. L. Frazer, and T. I. Baskin. Measurement of diffusion within the cell wall in living roots of arabidopsis thaliana. *J Exp Bot*, 58(11):3005–15, 2007.

[7] E. M. Kramer, H. L. Rutschow, and S. S. Mabie. Auxv: a database of auxin transport velocities. *Trends in Plant Science*, 16(9):461 – 463, 2011.

[8] N. L. Mellor, L. R. Band, A. Pěnčík, O. Novák, A. Rashed, T. Holman, M. H. Wilson, U. Voß, A. Bishopp, J. R. King, K. Ljung, M. J. Bennett, and M. R. Owen. Dynamic regulation of auxin oxidase and conjugating enzymes atDAO1 and GH3 modulates auxin homeostasis. *Proc Natl Acad Sci USA*, 113(39):11022–7, 2016.

[9] N. L. Mellor, U. Voß, G. Janes, M. J. Bennett, D. M. Wells, and L. R. Band. Auxin fluxes through plasmodesmata modify root-tip auxin distribution. *Development*, 147(6):dev181669, 2020.

[10] M. P. Pound, A. P. French, D. M. Wells, M. J. Bennett, and T. P. Pridmore. CellSeT: novel software to extract and analyze structured networks of plant cells from confocal images. *Plant Cell*, 24(4):1353–61, 2012.

[11] C. Pradal, S. Dufour-Kowalski, F. Boudon, C. Fournier, and C. Godin. OpenAlea: a visual programming and component-based software platform for plant modelling. *Functional Plant Biology*, 35(10):751–760, 2008.

[12] H. L. Rutschow, T. I. Baskin, and E. M. Kramer. Regulation of solute flux through plasmodesmata in the root meristem. *Plant Physiol*, 155(4):1817–26, 2011.

[13] A. N. Stepanova, J. Robertson-Hoyt, J. Yun, L. M. Benavente, D.-Y. Xie, K. Dolezal, A. Schlereth, G. Jürgens, and J. M. Alonso. TAA1-mediated auxin biosynthesis is essential for hormone crosstalk and plant development. *Cell*, 133(1):177–91, 2008.

[14] R. Swarup, E. M. Kramer, P. Perry, K. Knox, H. M. O. Leyser, J. Haseloff, G. T. S. Beemster, R. Bhalerao, and M. J. Bennett. Root gravitropism requires lateral root cap and epidermal cells for transport and response to a mobile auxin signal. *Nat Cell Biol*, 7(11):1057–65, 2005.

[15] W. Xuan, L. R. Band, R. P. Kumpf, D. Van Damme, B. Parizot, G. De Rop, D. Opdenacker, B. K. Möller, N. Skorzinski, M. F. Njo, B. De Rybel, D. Audenaert, M. K. Nowack, S. Vanneste, and T. Beeckman. Cyclic programmed cell death stimulates hormone signaling and root development in Arabidopsis. *Science*, 351(6271):384–7, 2016.

[16] T. Zhu, W. J. Lucas, and T. L. Rost. Directional cell-to-cell communication in the Arabidopsis root apical meristem i. an ultrastructural and functional analysis. *Protoplasma*, 203(1):35–47, 1998.

Supplemental Table S1: Cell sizes and numbers used in the geometrically regular template.

Measurement	Value	Measurement	Value
Stele width	5.9 μm	Regular cell length	14.6 μm
Number of stele files	6	Number of regular cells	30
Pericycle width	6.2 μm	Lateral root cap cell length	30.0 μm
Endodermis width	6.6 μm	Number of LRC cells	5
Cortex width	15.6 μm	Length of QC cells	6.1 μm
Epidermis width	12.2 μm	Length of columella initials	6.7 μm
LRC width	5.0 μm	Length of columella 1	5.6 μm
Length of columella 2	13.5 μm	Length of columella 3	14.4 μm
Length of columella 4	19.2 μm	Length of columella 5	24.5 μm

Supplemental Table S2: Primer pairs used for RT-qPCR (results of which are shown in Figure S11).

qABCB1$_f$	tcttccccgtagttgttgct
qABCB1$_r$	aaatgcagcaactgtcctga
qABCB4$_f$	ttagagccaacattgcttatgg
qABCB4$_r$	tttctcccaccatcgtatcat
qABCB19$_f$	agtcctcgctaactttgctca
qABCB19$_r$	ctttgcatgagccttagctgt
qACTIN2$_f$	ccgctctttctttccaagc
qACTIN2$_r$	ccggtaccattgtcacacac

Supplemental Table S3: Parameters for ABCB and PIN mediated efflux for each of the five ABCB-PIN interaction scenarios. The value used is based on the PIN permeability suggested in previous models (14).

Scenario	P_{PIN}	P_{ABCB}	P_{SYN}
I	0.56 μm s^{-1}	0.56 μm s^{-1}	0
II	0	0	0.56 μm s^{-1}
III	0.56 μm s^{-1}	0.56 μm s^{-1}	0.56 μm s^{-1}
IV	0	0.56 μm s^{-1}	0.56 μm s^{-1}
V	0.56 μm s^{-1}	0	0.56 μm s^{-1}

Supplemental Table S4: Model parameter values with associated references (where appropriate).

Parameter	Value	Reference
Permeabilities		
P_{IAAH}	0.56 μms^{-1}	(14)
P_{PIN}	0.56 μm s^{-1}	(14)
P_{AUX1}	0.56 μm s^{-1}	(14)
P_{LAX}	0.56 μm s^{-1}	Assumed same as P_{AUX1}
P_{plas}	0.806 μm^3 s^{-1}	(12), (16), (9)
Proportionality constants		
A_1	0.240	See (1) for derivation of all constants
A_2	3.56	
A_3	0.0.34	
B_1	0.004	
B_2	0.045	
B_3	4.67	
Apoplastic Diffusion		
D_{cw}	32 μm^2 s^{-1}	(6)
Apoplast thickness		
λ	0.14 μm	(5)
Auxin degradation		
β	0.001	As in (2)
Auxin production		
α_i^{HIGH} (QC, initials or 2 outer LRC layers)	0.01	(15; 13)
α_i^{LOW} (elsewhere)	0.001	As in (2)
DII-VENUS regulation		
p_1	0.06	(3)
p_3	0.91	(3)
p_4	0.03	(3)
